# Crustal weakening before rupture at volcanoes after long repose

**DOI:** 10.1038/s41467-026-77001-5

**Published:** 2026-08-24

**Authors:** Eric L. Newland, Christopher R. J. Kilburn

**Affiliations:** UCL Hazard Centre, Department of Earth Sciences, University College London, London, UK

**Keywords:** Volcanology, Natural hazards

## Abstract

At volcanoes reawakening after prolonged quiescence, local seismicity commonly increases before eruption. The earthquakes occur as crust extends over a zone of pressurising fluid several kilometres underground. The deformation causes existing faults to slip and new fractures to open. An eruption is most likely when the fractures grow large enough to rupture the crust. Fracture growth coincides with a net decrease in the elastic energy stored in the crust. Here we present a method for following changes in stored energy through a balance between the energy supplied by the pressure source and lost by seismicity. We test the method against unrest sequences at two large calderas, Sierra Negra and Campi Flegrei. The results provide a practical procedure for identifying the onset of fracture growth and, hence, for recognising an increased potential for crustal rupture.

## Abstract (web summary)

In this study, the authors show that accelerating volcano-tectonic earthquakes can occur as crustal stress decreases and fractures weaken the crust, providing a way to recognise when a reawakening volcano may be approaching rupture.

## Introduction

Volcanoes seal connections to their source magma during extended repose. Unrest returns when pressure increases in the trapped magma or a connected zone of magmatic fluids^1,2^. The crust extends over the pressure source, leading to detectable movement of the ground and the onset of small, local earthquakes or volcano-tectonic (VT) events^3–6^. Unrest is therefore accompanied by an increase in fracturing, raising two key questions: will this fracturing lead to rupture of the shallow crust, and will such rupture lead to an eruption?

Changes in local VT seismicity are commonly used to address these questions because they are readily detected, provide continuous records, and frequently show abrupt increases in event rate during the final approach to eruption. Numerous deterministic and probabilistic analyses have been developed to quantify the accelerations^7^. They all contain uncertainty and so, in spite of recent advances in deterministic modelling^8–10^, the alternative of empirically extrapolating accelerating rates of seismicity remains a standard procedure^11–15^.

By their nature, however, empirical analyses do not address the physics of unrest - in particular, how VT event rates reflect changes in the crust’s method for accommodating the strain energy supplied by a pressure source. Thus, approximating the crust to an elastic rock mass containing faults, the dominant mode of accommodating additional energy changes with deformation from elastic movement in unbroken volumes of rock, to elastic movement with fault slip, to fault slip, and finally to fault slip with fracture growth^8,9,16,17^. The full deformation sequence is associated with increasing rates of fault slip, and hence rates of VT seismicity, until rupture^8,11^.

Conventional interpretations assume that faster VT event rates require the strain energy stored in the crust to increase or remain constant^8^. Here, we argue that, contrary to expectation, fracture-induced weakening of the crust can culminate in rupture even when the amount of stored energy is decreasing. We have developed a practical method for measuring changes in the strain energy stored in deforming crust by calculating the difference between the energy supplied by a pressurising source and the amount lost through VT earthquakes (see ‘Methods’). The stored energy yields a scalar measure of the mean differential stress in the crust and, by tracking the variation of this stress with time, we can identify the transition from deformation dominated by increasing stress to that dominated by decreasing crustal strength. We have tested our method against time series of unrest at two closed-system calderas, a pre-eruptive sequence from Sierra Negra (Galapagos Islands) and a continuing sequence at Campi Flegrei (southern Italy).

Here we show that (1) VT event rates continue to increase under a decreasing stress in a manner independent of commonly-invoked destabilising processes—such as increases in pore-fluid pore pressure or the viscous deformation of high-temperature crust; and (2) fracturing promotes accelerations in ground uplift even when the rate of source pressurisation remains unchanged. Both results are consistent with the fracture-induced weakening of the crust. The emergence of increasing VT event rate under decreasing stress is thus a strong indicator that fractures may be approaching the conditions necessary for opening a new pathway in the crust ahead of an eruption.

## Results

Among volcanoes reawakening after long repose, unrest sequences at large calderas are typically ten or more times longer than those commonly observed at stratovolcanoes^18,19^. They are thus ideal for following detailed changes with time in how the stored energy and mean stress evolve in volcanic crust. For our analysis, we have selected unrest from Sierra Negra and Campi Flegrei calderas as examples of sequences that, respectively, culminated in an eruption and remain in progress.

### Sierra Negra: 2005–2018

Sierra Negra is a large basaltic shield volcano on Isabela Island, Galápagos, Ecuador. It is the most voluminous of the Galápagos volcanoes and was constructed by successive eruptions of basaltic lava flows^20^. The summit is occupied by a shallow trapdoor caldera, approximately 7.2 by 9.3 km in diameter, bounded by near-vertical ring faults^21^. The caldera contains a well-documented resurgent block, interpreted to reflect repeated uplift of the caldera floor during episodes of magma accumulation beneath the summit^22^. Surface deformation is thought to be driven primarily by a shallow sill-like source at a depth of around 2 km, fed by a deeper magmatic system^21^. Hydrothermal activity is comparatively localised, but persistent fumarolic degassing at Minas de Azufre indicates fluid ascent focused along the caldera fault system^23^.

Sierra Negra has had at least seven eruptions in the past century^24,25^. The most recent occurred in 2018 after 13 years in repose and lasted for 58 days. Prior to the eruption, ground deformation had been monitored by a continuous GPS network^25^ and, from 2009, seismicity had been recorded by a network of six permanent seismometers^21,26^.

**Fig. 1:**
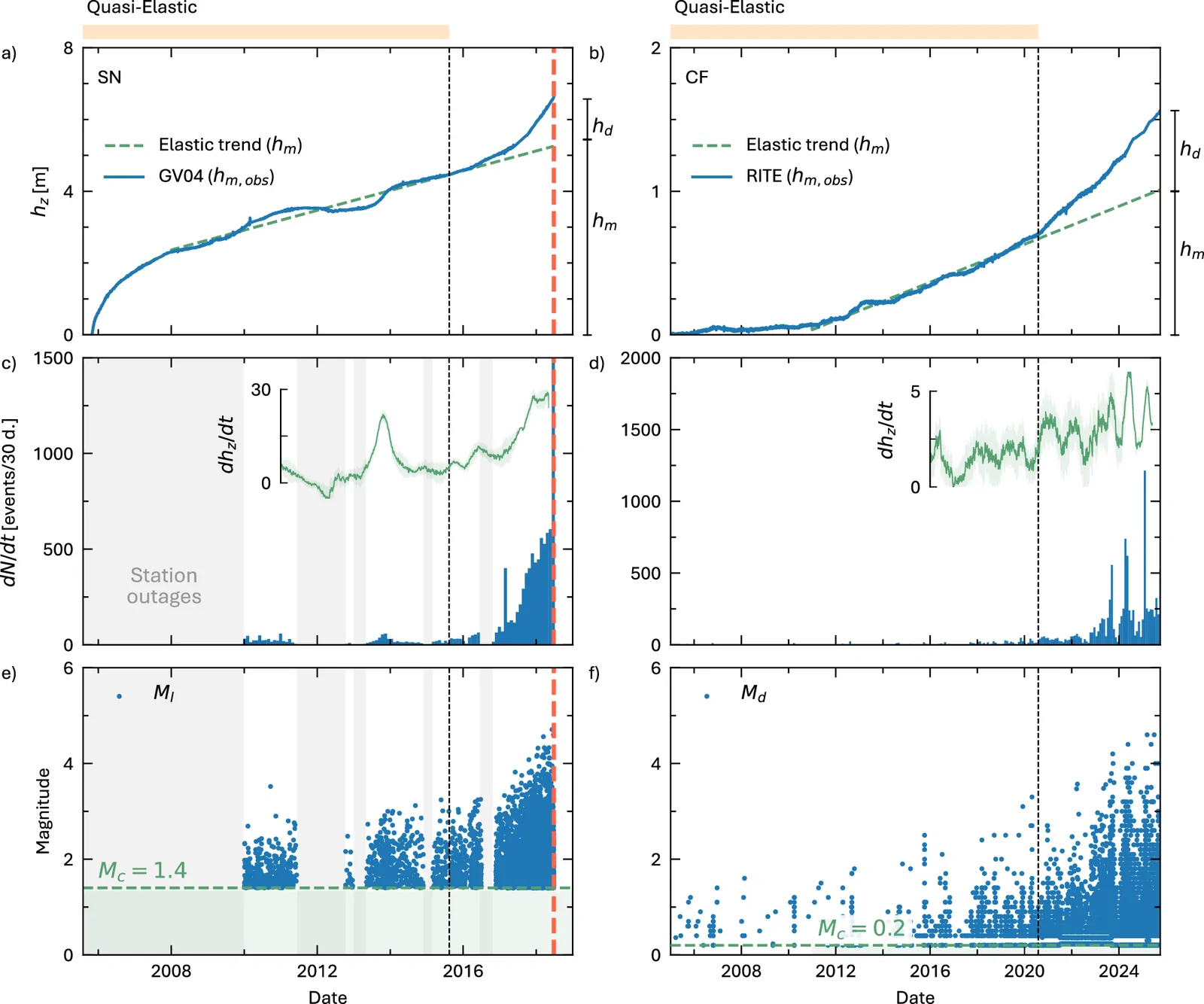
Key geophysical time series for Sierra Negra and Campi Flegrei. The left column shows data for Sierra Negra and the right column shows data for Campi Flegrei. **a**, **b** Maximum vertical uplift, h (blue), and the pre-seismic linear uplift trend (teal dashed). h and h denote the maximum observed uplift and the maximum uplift predicted from source-pressure changes, respectively. **c**, **d** VT seismic event rate, dN/dt, expressed as the number of events per 30 days. Insets show the uplift rate in mm day. **e**, **f** Earthquake magnitude through time (Sierra Negra: local magnitude M; Campi Flegrei: duration magnitude M); dashed horizontal lines indicate the magnitude of completeness M. Periods of missing seismic data at Sierra Negra are shown by grey shaded bands (station outages;^21^). The red dashed line marks the eruption date (Sierra Negra). Beige bars indicate the quasi-elastic interval; black dashed vertical lines mark regime boundaries.

**Fig. 2:**
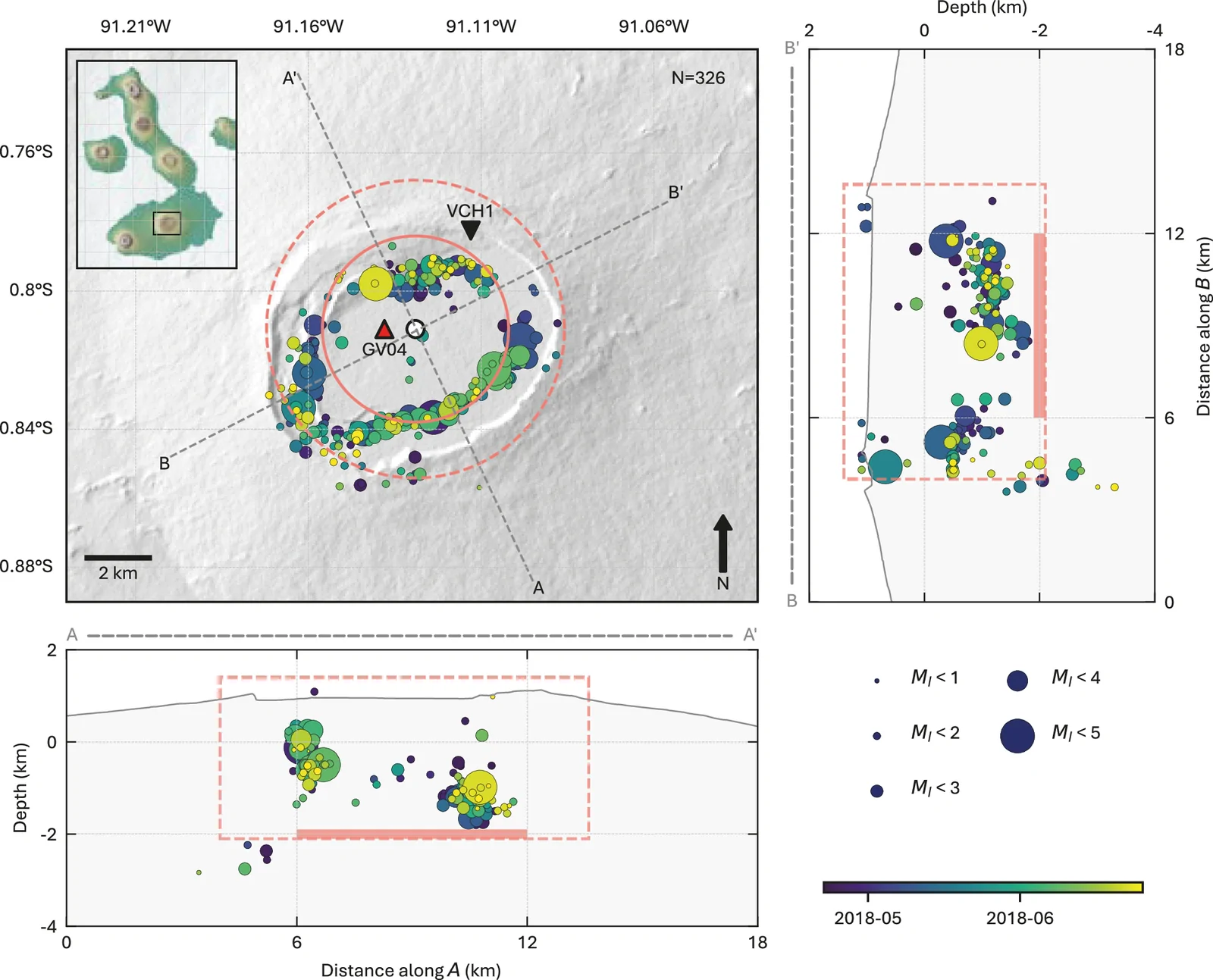
Spatial distribution of earthquake hypocentres at Sierra Negra from 2018-04-22 to 2018-06-26 (eruption date; *N* = 326). Circles show individual events, coloured by occurrence date and scaled by magnitude. Dashed grey lines in map view mark the traces of cross-sections $A-{A}^{{\prime} }$ and $B-{B}^{{\prime} }$. Cross-sections include events within 2 km perpendicular distance of each profile. The solid red circle in plan view and solid red rectangles in the cross-sections indicate the inferred pressure source geometry ^27^. Dashed red outlines (circle in plan view; rectangles in cross-sections) delineate the inferred deforming volume, *V*_*d*_ (Supplementary Information B). Triangles mark the locations of GPS station GV04 (red) and permanent seismic station VCH1 (black).

Following subsidence during the 2005 eruption, surface uplift began in October 2005. The maximum uplift was recorded at the centre of the caldera at station GV04^25^. An uplift of 1.77 m occurred between October 2005 and June 2007, after which it continued at a steady rate of about 0.3 m yr^−1^ until 2016 (Fig. 1). From 2016 the uplift accelerated and reached a peak rate of about 1.4 m yr^−1^ before an eruption began on 26 June 2018, when the total uplift had reached about 6.5 m (Fig. 1). Considered across the whole period from 2005 to 2018, the increase in ground deformation is consistent with the inflation of a shallow sill-like source at a depth of 2.1 km and with an average radius of about 3 km^25,27^. Figure 1 shows the VT seismic event rate recorded by stations GS09 (2009–2011) and VCH1 (2012–present), installed on the north rim of the summit caldera in 2012 as part of the new monitoring network (Fig. 2;^25,26^). Local earthquake magnitudes, *M*_*l*_, were estimated using a completeness magnitude, *M*_*c*_, of 1.4^25^. Between 2012 and mid-2016, the VT event rate remained low, averaging approximately 40 events per month. The rate began to accelerate rapidly in 2016 and reached approximately 500 events per month by 2018. It then continued to increase more gradually until the eruption began on 26th June. The maximum magnitude *M*_*l*_ remained at about 3.3 from 2012 to 2017, after which it increased in coincidence with the acceleration in VT event rate and surface deformation. The increase was approximately linear and reached a maximum value of 4.7 on the day of the eruption.

Earthquake hypocentres are available from late April 2018, when the IGUANA campaign network was installed^21^. They show that most seismicity occurred at depths of 3 km or less and was restricted to the rim of the caldera (Fig. 2), around an inferred trapdoor fault^21,22,25^. They also lacked any systematic changes with time.

### Campi Flegrei: 2005–2025

The Campi Flegrei volcano is a large caldera, about 14 km across, that lies on the western side of Naples in southern Italy^28^. Owing to its high population, approaching 500,000 people^29^, and its potential for explosive volcanism^28,30,31^, it is today one of the most closely monitored volcanoes on Earth^32,33^.

Beneath Campi Flegrei lies a melt-rich zone that extends upwards to a depth of approximately 8 km^34^. This deep source intermittently feeds a shallow sill-like structure at a depth of 3 km, which is inferred to account for most of the surface deformation observed in recent history^35^. The shallow crust above this structure consists predominantly of primary and reworked pyroclastic volcanic deposits overlying a horizon of thermally altered marine silts and clays^36^. The caldera is host to a complex system of faults, associated with the formation of the caldera and the subsequent deformation^37^. The volcano is also characterised by a vigorous hydrothermal system that extends to depths of about 2.5–3 km^38^.

At least 70 eruptions^39^ have occurred across the floor of the caldera since its latest^40^, or possibly only^41^, episode of caldera collapse c.15 ka BP. The majority have been explosive with volumes ~0.01−1 km^3^ (DRE). The only historical eruption occurred in 1538 to create the cone of Monte Nuovo^42,43^. The eruption was followed by four centuries of caldera-wide subsidence, with a maximum of 8–9 m near Pozzuoli, a coastal town near the centre of the volcano^44,45^. Subsidence changed to uplift around 1950, with the growth of a radially-symmetric bulge about 5 km in radius centred near Pozzuoli^33^. Since then, Pozzuoli has been raised intermittently by a total of about 4.3 m: c. 0.8 m in 1950–1952, c. 1.75 m in 1969–1972, c. 1.75 m in 1982–1984 and c. 1.45 m between 2005 and May 2025, set against a cumulative subsidence of about 1.3 m during the intervals in between^33,46^.

This study focuses on Campi Flegrei’s most recent episode of unrest, which began in 2005^47^. For comparison with the unrest at Sierra Negra in 2005–2018, maximum uplift has been used as a measure of ground deformation. The maximum uplift was recorded at Station RITE in the Pozzuoli’s historical centre, Rione Terra (Fig. 1; see ref. ^33^). Starting in 2005, the total uplift at Campi Flegrei reached approximately 0.05 m by 2011 and 0.65 m by 2020. To a first approximation, the uplift rate between 2011 and 2020 was nearly constant at about 0.07 m yr^−1^. After 2020, the uplift rate exhibited periodic accelerations, returning to the pre-2020 rate in the intervening periods. As in the preceding episodes of unrest, the ground uplift has developed as a radially symmetric bulge about 5 km in radius around Pozzuoli^33^, consistent with the inflation of a sill-like pressure source at depths 3–4 km^35,48,49^.

**Fig. 3:**
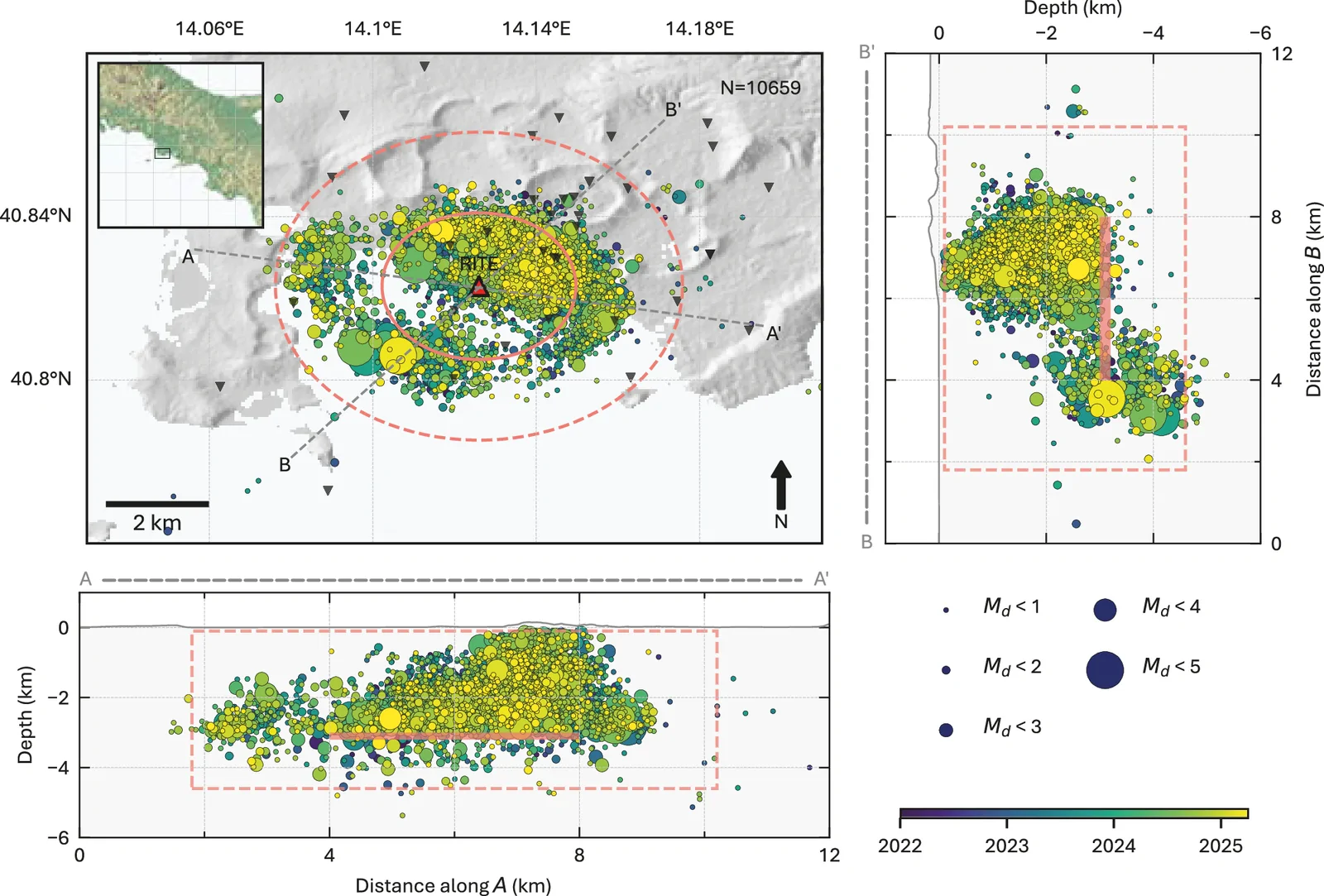
Spatial distribution of earthquake hypocentres at Campi Flegrei from 2022 to 2025-10 (*N* = 10,659). Circles show individual events, coloured by occurrence date and scaled by magnitude. Dashed grey lines in map view mark the traces of cross-sections *A*-${A}^{{\prime} }$ and *B*-${B}^{{\prime} }$. Cross-sections include events within 2 km perpendicular distance of each profile. The solid red circle in plan view and solid red rectangles in the cross-sections indicate the inferred pressure source geometry ^35^. Dashed red outlines (circle in plan view; rectangles in cross-sections) delineate the inferred deforming volume, *V*_*d*_ (Supplementary Information B); the plan-view circle appears elliptical due to the Plate Carrée projection. The red triangle marks the GPS station RITE and black inverted triangles mark permanent seismic stations.

Most of the contemporaneous VT seismicity has occurred at depths of less than 3 km and within the northeastern sector of uplift, covering Pozzuoli and the neighbouring residential areas of Bagnoli and Agnano to the east; a second cluster of hypocentres follows an arcuate NW-SE trend offshore about 2 km SW of Pozzuoli (Fig. 3; see ref. ^32^). Between 2005 and 2020, the VT event rate remained low, averaging at about 2 events per month (Fig. 1). The rate increased to an average of about 32 events per month between 2020 and 2022, before escalating rapidly during 2022–2025 and culminating in three major seismic swarms—in September-October 2023, April 2024 and February 2025—with a maximum rate of 1212 events per month in February 2025. The size of VT events at Campi Flegrei is measured by their duration magnitude, *M*_*d*_, with a completeness value *M*_*c*_ of 0.2. Since 2011, the maximum magnitude has increased approximately linearly with time, from about 1.8 to a peak of 4.6 in March 2025.

### Strain energy and stored stress in the crust

We used monitoring data from Sierra Negra and Campi Flegrei to reconstruct how the mean differential stress within their crusts has varied with time. Increases in source pressure deform the overlying crust, increasing the effective differential stress and thereby storing elastic strain energy. At volcanoes, crustal deformation occurs predominantly through changes in shape of the crust rather than changes in its volume^2^. Elastic strain energy is therefore stored mainly as distortional (shear-related) strain energy, with a smaller contribution from volumetric (isotropic-related) strain energy (Supplementary Information B). Part of this stored distortional energy is released through VT earthquakes. The resulting history of mean differential stress is therefore determined by the balance between elastic strain energy supplied by the pressure source and that dissipated through seismicity.

The total energy supplied to the crust is given by the mechanical work done by the pressurising source during deformation. This work is proportional to the product of source pressure and the resulting change in source volume (see ‘Methods’; see ref. ^50^). We estimate this energy input using an elastic source model that relates the observed uplift history to source overpressure and volume change. Assuming that source work is converted predominantly into distortional elastic strain energy, rather than volumetric strain energy or viscous dissipation (Supplementary Information E), the resulting estimate provides an upper bound on the distortional elastic strain energy stored in the crust. Elastic strain energy dissipated by earthquakes is estimated using the event magnitudes together with an empirically constrained seismic efficiency (see ‘Methods’; see ref. ^51^).

The mean differential stress associated with the remaining stored elastic strain energy is then obtained by relating the distortional elastic energy density to stress (Methods):

1 $${\sigma }_{d}(t)=\sqrt{6G\frac{\overline{U}(t)}{{V}_{d}}}.$$

Here, $\overline{U}(t)$ is the stored distortional elastic strain energy, *G* is the mean shear modulus of the crust, and *V*_*d*_ is the characteristic volume over which deformation is distributed. Throughout this study, -mean stress- refers to the spatially averaged scalar measure of differential (deviatoric) stress rather than to the full stress tensor, and therefore captures changes relative to the energy stored at the beginning of the analysis. This quantity is equivalent to the von Mises stress, which is commonly used as a failure criterion^52^.

The patterns of ground movement at both Sierra Negra (Fig. 2) and Campi Flegrei (Fig. 3) are consistent with sill-like pressure sources^25,35^. For these geometries, we use the penny-shaped crack model in a homogeneous elastic half-space^53^ to relate maximum surface uplift to source overpressure and source volume change, and hence estimate the elastic strain energy transferred to the crust (see ‘Methods’ Eq. (5)). The deformation model assumes the crust is linearly elastic^53^, for which uplift is proportional to source pressure. In particular, the maximum vertical uplift *h*_*m*_ is given by the general formula:

2 $${h}_{m}=C\left(1-\nu \right)\left(\frac{P}{G}\right){r}_{0}{\left(\frac{{r}_{0}}{{z}_{0}}\right)}^{m}$$

where *P*, *r*_0_, and *z*_0_ are the source overpressure, radius and depth, *ν* is Poisson’s ratio and *G* is the shear modulus of the crust. The constants *C* and *m* depend on the ratio *r*_0_/*z*_0_: when *r*_0_/*z*_0_ ≪ 1, *C* = 4/*π* and *m* = 2^53^, but when *r*_0_/*z*_0_ ≈ 1, *C* = 8/3*π* and *m* = 5/3^54^.

**Fig. 4:**
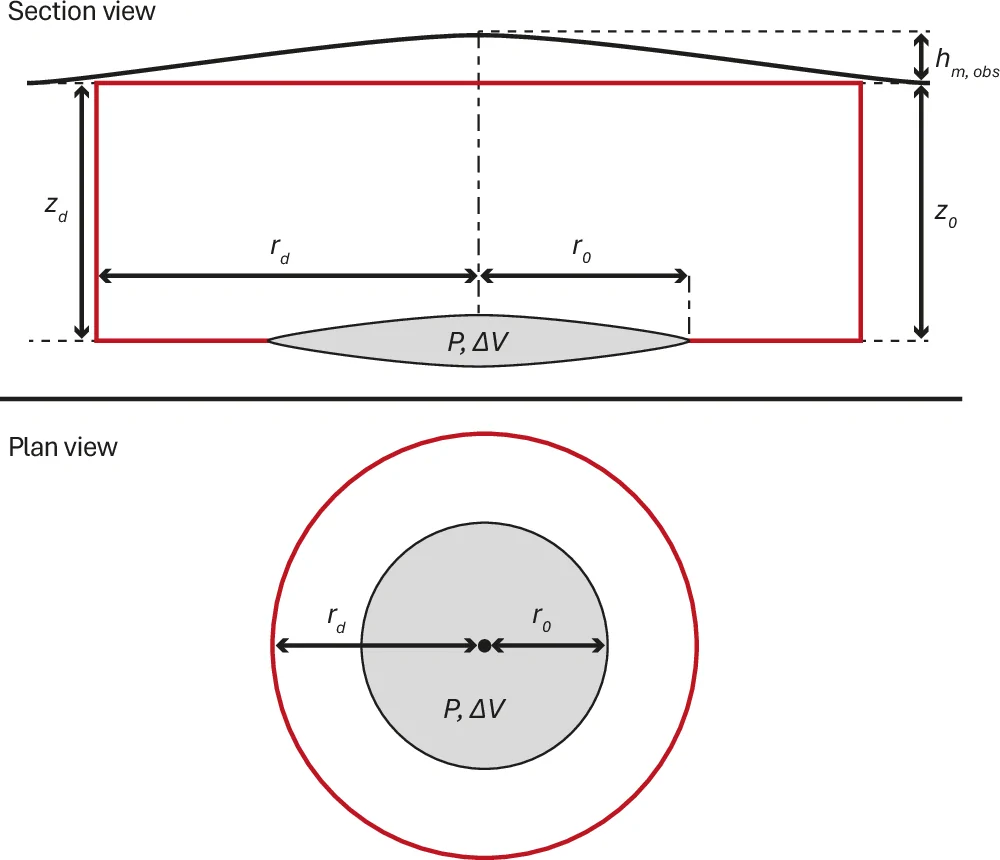
Schematic of the sill-source geometry and crustal deformation volume used to estimate elastic strain energy. A sill-like pressure source of radius *r*_0_, overpressure *P*, and volume change Δ*V* is located at depth *z*_0_ beneath the deforming crust. The resulting surface uplift has maximum amplitude *h*_*m*,obs_. The red boundary marks the cylindrical volume of deformation within which elastic strain energy is stored, with a radius *r*_*d*_ and vertical extent *z*_*d*_. Left, section view; right, plan view.

Assuming that the sources are axisymmetric, we approximate the crustal volume in which the elastic strain energy is stored (*V*_*d*_) as a cylinder centred on the source, with radius *r*_*d*_ and depth *z*_*d*_ (see ‘Methods’, Eq. (12); Figs. 2–4). We set *z*_*d*_ = *z*_0_, and compute *r*_*d*_ from the penny-shaped crack model as the radius enclosing 99% of the elastic strain energy. Values of the deformation radius *r*_*d*_ are derived in Supplementary Information B. The modelling parameters are summarised in Fig. 4 and Table 1.

**Table 1:** Source parameters used in the Fialko model

Parameter	Campi Flegrei	Sierra Negra	Unit
Source radius, *z*_0_	3.0	2.1	km
Source radius, *r*_0_	2.0	3.0	km
Deformation depth, *z*_*d*_	4.6	3.6	km
Deformation radius, *r*_*d*_	3.0	6.0	km
Shear modulus, *G*	7	16	GPa
Poisson’s ratio, *ν*	0.25	0.25	n.a.

At Sierra Negra, we adopt a source geometry with radius 3000 m and depth 2100 m in a homogeneous elastic half-space, consistent with previous geodetic models^25,27,55^ (Fig. 2). We set *G* = 16 GPa, consistent with reported values for basalt^22^. At Campi Flegrei, we adopt the source geometry inferred by ref. ^35^, who show that a homogeneous elastic model adequately reproduces the observed deformation and infer a source depth of 3000 m and radius of 2000 m. We set *G* = 7 GPa, representing an average for the shallow layered crust based on seismic tomography^56,57^. Departures from homogeneity in the crust are incorporated as uncertainty in the effective shear modulus of the deforming volume and are propagated through the error analysis (Supplementary Information D).

The value of the shear modulus is influenced by pre-existing discontinuities in the crust. Field-scale measurements, such as seismic tomography, provide direct estimates of the shear modulus *G*, inherently incorporating the effects of these discontinuities. As deformation progresses, additional discontinuities develop, further reducing the modulus and weakening the mechanical resistance of the crust. This weakening is represented by a damage variable *D*, which quantifies the extent of additional damage such that the effective modulus ${G}^{{\prime} }=G(1-D)$. Thus, ${G}^{{\prime} }$ represents the modulus including the effects of pre-existing discontinuities and additional damage^58,59^, so that *D* = 0 corresponds to the initial state of the crust and *D* = 1 corresponds to a completely ruptured crust.

The maximum observed surface uplift, *h*_m,obs_, is the sum of the uplift due to elastic stretching of the crust, *h*_m_, and the uplift associated with increasing damage, *h*_d_. As deformation continues, damage accumulates, increasing the amount of deformation produced by a given increase in source pressure. As a result, the proportion of total uplift attributable to damage also increases.

Because damage cannot be observed directly, its influence on surface uplift must be inferred from contemporaneous changes in uplift and VT seismicity. At both calderas, deformation begins with an initial adjustment phase (Sierra Negra, 2006–2008; Campi Flegrei, 2005–2011; Fig. 1), followed by a prolonged interval of near-constant uplift rate (Sierra Negra, 2008–2015; Campi Flegrei, 2011–2020; Fig. 1). During these intervals, VT seismicity remains low and event rates increase gradually. At Sierra Negra, we assume that seismicity rates during outages in the early phases were similar to those recorded immediately before and after each outage and therefore remained low. In practice, the amounts of seismicity during these periods are small enough for the evolving behaviour to be approximated to single elastic trends, so that the constant rate of observed uplift indicates a constant rate of pressure increase in the source. We refer to this deformation regime as quasi-elastic, which means that elastic deformation predominates despite the presence of minor inelastic processes.

Rates of uplift accelerated above their previously established near-constant values with the onset of increased seismicity, marking the end of quasi-elastic deformation (Fig. 1). We estimate the timing of these increases using a simple sliding-window analysis applied to both the vertical uplift time series and the cumulative number of VT events (Supplementary Information A). Near-synchronous step increases in uplift and VT seismicity rates are identified at both volcanoes, occurring in August 2015 at Sierra Negra and August 2020 at Campi Flegrei (Fig. 1). At Sierra Negra, the acceleration in seismicity is sustained and is mirrored by a steadily increasing uplift rate. At Campi Flegrei, by contrast, seismicity occurs in swarms. This episodic behaviour is reflected in the uplift record, with the uplift rate increasing during periods of seismicity and returning, during intervening quiescent periods, to the rate characteristic of the quasi-elastic regime.

The near-synchronous increases in the rates of uplift and seismicity at both calderas can be explained by two end-member mechanisms: an increase in the rate of source pressurisation or the onset of significant crustal weakening. The first interpretation requires pressurisation to have increased at both volcanoes for unspecified reasons; the second follows the natural behaviour of crustal fracturing under increasing deformation at any volcano^8,9,17,60–62^.

We adopt the simpler and more general interpretation that crustal fracturing controlled the increase in uplift rate above that expected from source pressurisation alone. That is, we assume that the observed uplift acceleration was caused by increasing damage in a crust subjected to an otherwise unchanged constant pressurisation rate. Accordingly, we use intervals of near-constant uplift during periods of low VT seismicity (Sierra Negra: 2008–2016; Campi Flegrei: 2011–2020) to calibrate a baseline source pressurisation rate. We then extrapolate this baseline forwards in time using our elastic source model to define a reference uplift time series and energy-input history for the case of linearly increasing source pressure in the absence of additional crustal damage. Figure 1 compares the observed uplift histories (*h*_*m*,obs_) with the uplift histories (*h*_*m*_) predicted for a constant pressurisation rate and no additional crustal damage. Because the inferred pressurisation history is not unique, particularly at Campi Flegrei, we assess the sensitivity of assuming a constant rate of pressurisation in Supplementary Information C by repeating the analysis using alternative power-law fits to the uplift time series. This test shows that the resulting temporal evolution of elastic strain energy and inferred regime transitions are insensitive to the assumed uplift parameterisation.

To evaluate whether the magnitude of the additional uplift can reasonably be associated with VT seismicity, we compare the geodetic moment associated with the additional uplift attributed to damage (*h*_*m*,obs_ − *h*_*m*_) with the seismic moment released over the same interval. If the uplift acceleration is driven by seismicity-induced damage, these two moments should be similar in magnitude. For a planar source such as a sill-shaped source, the geodetic moment is given by ref. ^63^:

3 $${M}_{g}=G\Delta {V}_{o},$$

where *G* is the shear modulus and Δ*V*_*o*_ is the volume of sill-shaped opening associated with the uplift due to an increase in damage; we estimate Δ*V*_*o*_ using the Fialko model. The cumulative seismic moment, Σ*M*_*o*_ is calculated from the earthquake catalogue using the standard moment-magnitude relation^64^. At Sierra Negra, we obtain *M*_*g*_ ≈ 3.9 × 10^17^ Nm and Σ*M*_*o*_ ≈ 1.7 × 10^17^ Nm; at Campi Flegrei, *M*_*g*_ ≈ 9.9 × 10^17^ Nm and Σ*M*_*o*_ ≈ 3.3 × 10^17^ Nm. In both cases, the geodetic moment associated with the additional uplift and seismic moments agree to within a factor of ~2−3 (i.e. the same order of magnitude) supporting the interpretation that the additional uplift during these intervals can be attributed to contemporaneous seismicity.

**Fig. 5:**
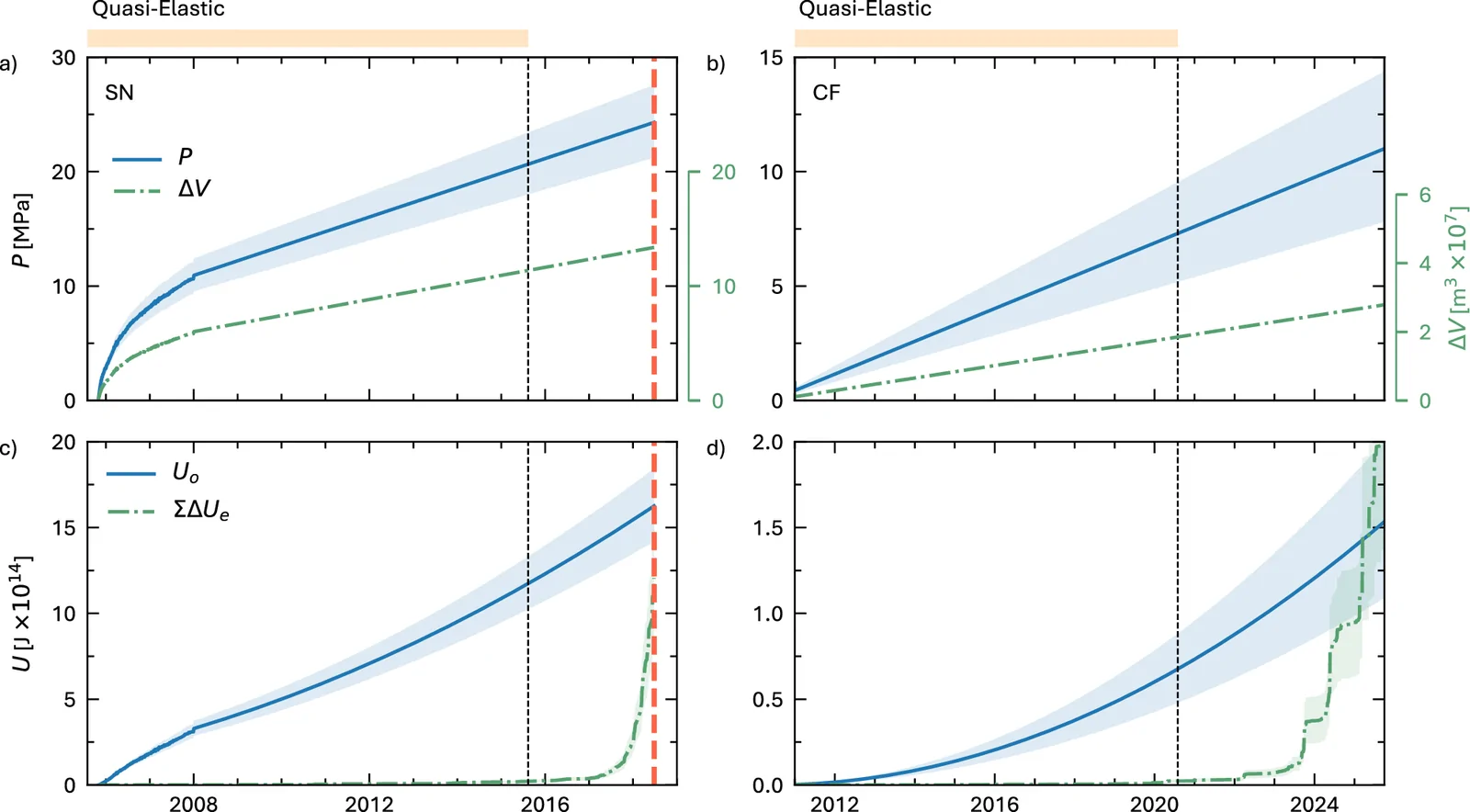
Time evolution of source overpressure, volume change and elastic strain energy. **a**, **c** Sierra Negra (2005–2019) and (**b**, **d**) Campi Flegrei (2011 to 10-2025) . **a**, **b** Source overpressure, *P* (blue solid line), and source volume change, Δ*V* (teal dot-dash line), estimated from the linear uplift trend during the quasi-elastic period using the parameters in Table 1. **c**, **d** Mean elastic strain energy transferred to the crust, *U*_*o*_ (blue solid line), and cumulative elastic strain energy loss, ∑*ΔU*_*e*_ (teal dot-dash line), through time. Shaded envelopes show model uncertainty as described in Supplementary Information D. Vertical dashed lines mark the end of the quasi-elastic period; the red dashed line marks the eruption date at Sierra Negra. Beige bars indicate the quasi-elastic period.

The uplift trend associated with a constant pressurisation rate and no additional damage, *h*_*m*_, together with the penny-shaped crack model, yields estimates of source overpressure. Together with Eq. (10) and the modelling parameters in Table 1, these estimates are used to reconstruct the temporal evolution of elastic strain energy transferred to the crust at the two volcanoes (Fig. 5).

The contemporaneous loss of energy through VT seismicity was calculated using Eq. (7) together with the number and magnitudes of earthquakes recorded at Sierra Negra^25^ and Campi Flegrei^32^. At Sierra Negra, we assumed that the measured local magnitude was equivalent to the moment magnitude, *M*_*w*_^25^. At Campi Flegrei, we converted the measured duration magnitude, *M*_*d*_, to *M*_*w*_ using the local conversion function from^65^. The calculated seismic energy was converted to an equivalent drop in strain energy using the seismic efficiency, *η*.

Figure 5 shows the resulting mean rates of elastic strain energy release, using *η* values compiled by ref. ^66^ for small-magnitude (*M*_*w*_ < 3.3) mining-induced earthquakes, comparable to the VT events considered here. Uncertainty associated with *η* (and the resulting energy-release estimates) is propagated using the Monte Carlo approach described in Supplementary Information D. Both volcanoes exhibit rapid increases in energy release following the initial quasi-elastic phase of deformation.

**Fig. 6:**
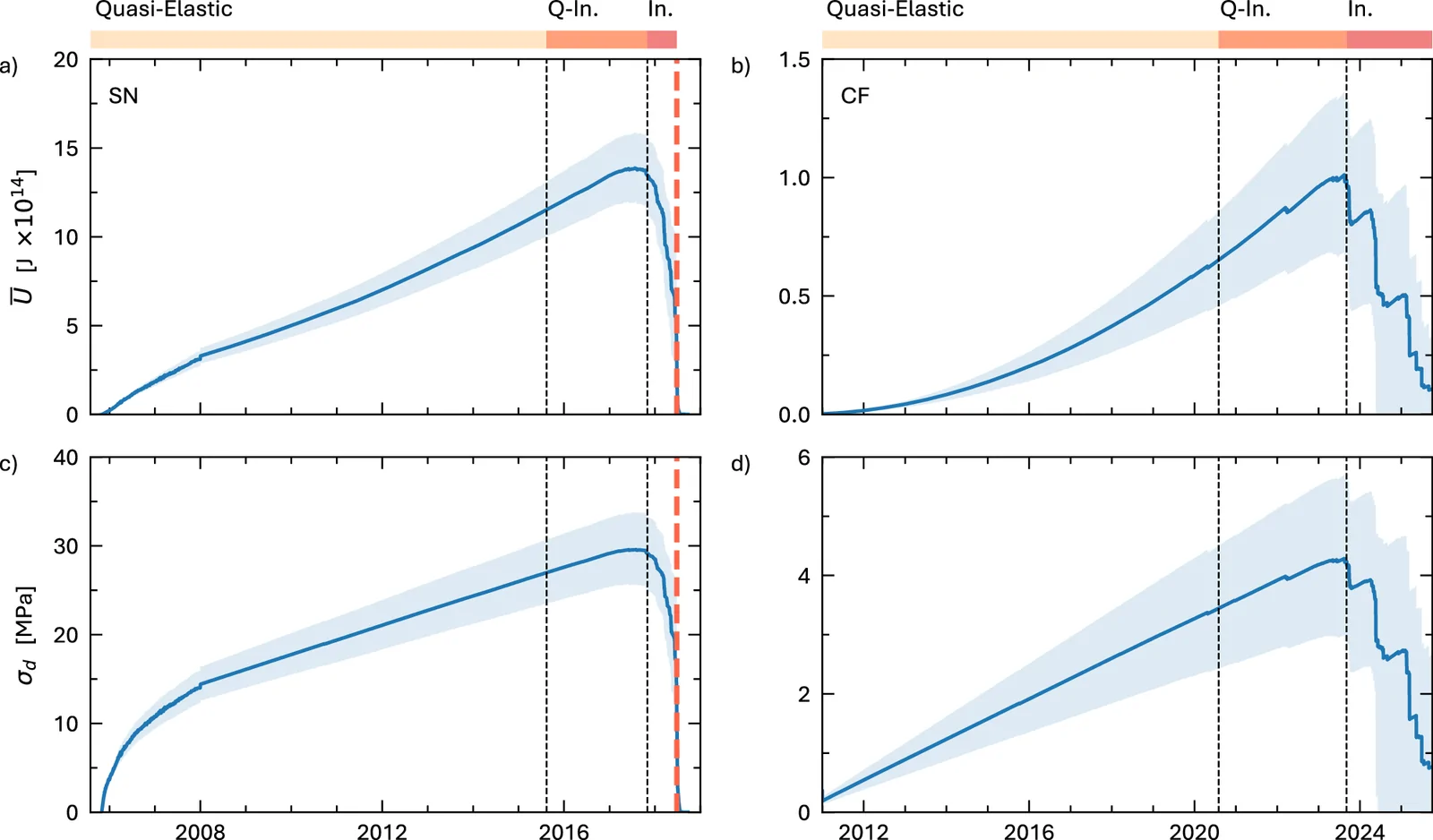
Time evolution of resultant elastic strain energy and mean differential stress. **a**, **c** Sierra Negra (2005–2019) and (**b**, **d**) Campi Flegrei (2011 to 10-2025) . **a**, **b** Mean elastic strain energy, $\bar{U}$, stored in the crust through time, and (**c**, d) mean differential stress, *σ*_*d*_, through time. Shaded envelopes show model uncertainty as described in Supplementary Information D. Beige, orange, and red bars indicate the quasi-elastic, quasi-inelastic, and inelastic regimes, respectively; vertical black dashed lines mark regime boundaries. The vertical red dashed line marks the eruption date at Sierra Negra.

Figure 6 shows the evolution of elastic strain energy stored in the crust, expressed as the ensemble mean and standard deviation from the Monte Carlo uncertainty analysis presented in Supplementary Information D. Stored energy is calculated as the difference between the energy transferred to the crust by source pressurisation and the energy released through seismicity (Eq. (10)). In both cases, stored energy increases until the rate of energy loss becomes comparable to the rate of supply, yielding peak mean stored energies of 1.39 × 10^15^ J in October 2017 at Sierra Negra, and 1.01 × 10^14 ^J in August 2023 at Campi Flegrei. Subsequently, the rate of stored energy decreases more abruptly than its earlier rate of increase (Fig. 6).

The mean differential stress in the crust is proportional to the square root of stored energy (Eq. (12)) and, hence, also shows an overall increase and then decrease with time (Fig. 6). The behaviour is clearly seen using *σ*_*d*_ as a measure of mean differential stress (Eq. (12)). Thus, *σ*_*d*_ increased at an approximately constant rate during the initial quasi-elastic period (and so consistent with the approximately constant rate of uplift), reaching maximum values of 29.5 MPa at Sierra Negra and 4.15 MPa at Campi Flegrei (Fig. 6), coinciding with the date of maximum stored energy. Although absolute stress magnitudes are sensitive to uncertainty in elastic properties and seismic efficiency, the temporal evolution and timing of peak stress are robust across the ensemble runs in our error analysis (Supplementary Information D).

### Characteristics of earthquake populations

**Fig. 7:**
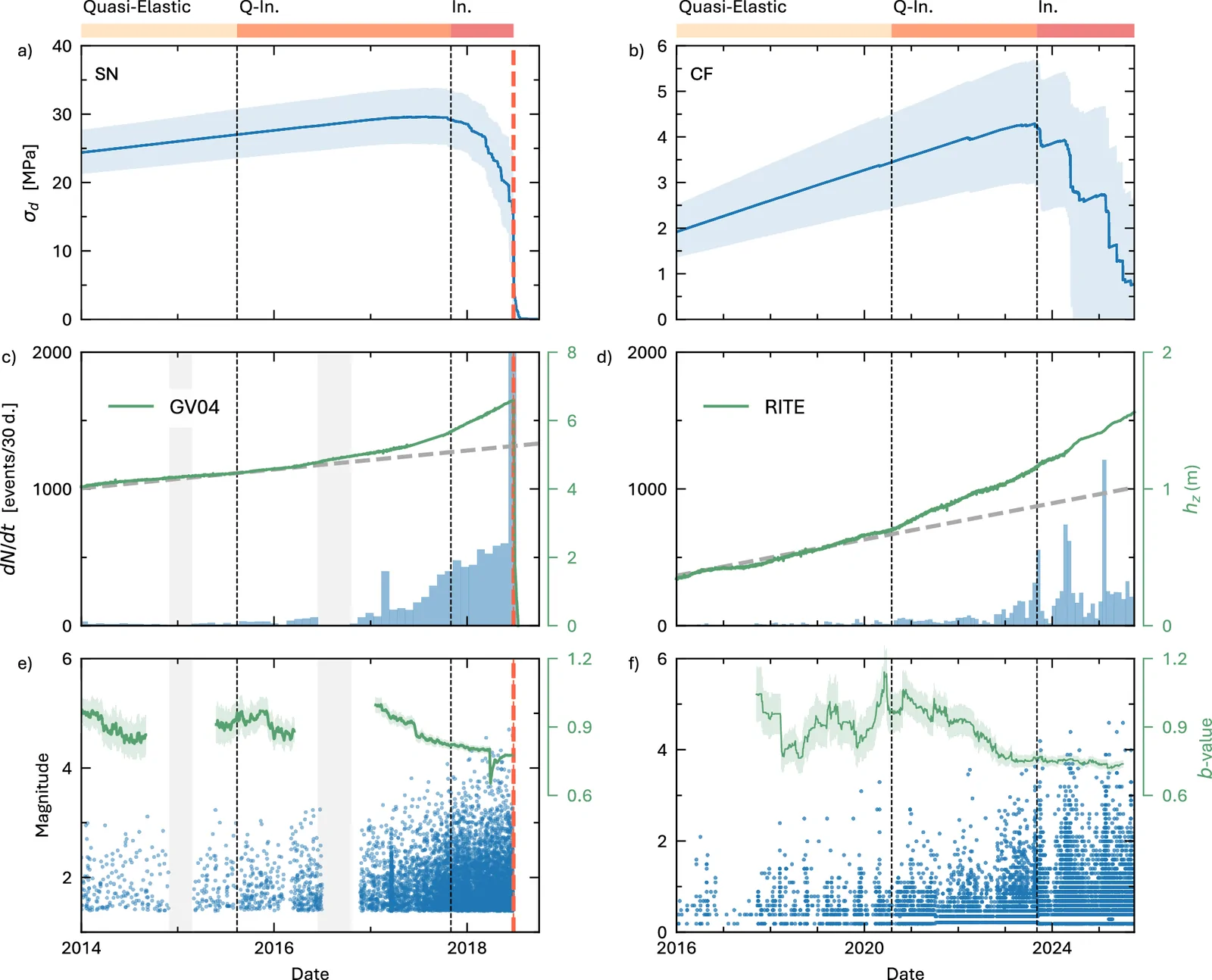
Time evolution of mean differential stress, seismicity and vertical uplift. **a**, **c**, **e** Sierra Negra (2014-2019) and (**b**, **d**, **f**) Campi Flegrei (2016 to 10-2025)-). **a**, **b** Mean differential stress, *σ*_*d*_ (MPa), through time. **c**, **d** Left axis: seismic event rate, *dN*/*dt*, shown as events per 30 days; right axis: maximum vertical uplift, *h*_*z*_ (m). **e**, **f** Left axis: event magnitude, *M*; right axis: seismic *b*-value computed using a 6-month rolling window. Grey shaded bands in the Sierra Negra panels indicate periods of missing seismic data. Beige, orange, and red bars indicate the quasi-elastic, quasi-inelastic, and inelastic regimes, respectively; vertical black dashed lines mark regime boundaries. The vertical red dashed line marks the eruption date at Sierra Negra.

The characteristics of the earthquake populations also show similar temporal variations at Sierra Negra and Campi Flegrei. Maximum earthquake magnitudes increase gradually throughout the period of analysis (Fig. 7). The corresponding seismic b-values (see ‘Methods’), which describe the frequency-magnitude distributions of earthquakes, show no clear systematic trend until the end of the quasi-elastic regime, after which they decrease steadily towards a near-constant value.

At Sierra Negra, most earthquakes during the quasi-elastic regime between 2014 and mid-2016 had *M*_*l*_ < 2.5, although occasional events reached *M*_*l*_ = 3.5. Between 2017 and 2018, the maximum magnitude increased approximately linearly from about *M*_*l*_ = 3.0–4.7 (Fig. 7). The mean b-value remained close to 0.9 until mid-2016 and subsequently decreased from about 1.0–0.8 during 2017–2018, with a temporary decline to 0.65 in early 2018 (Fig. 7)^21^. At Campi Flegrei, most VT events during quasi-elastic deformation had *M*_*d*_ < 2.5, although occasional events reached *M*_*d*_ = 3.0. After 2020, the maximum magnitude increased at an approximately constant rate from about *M*_*d*_ = 2.5 to 4.6 by 2025 (Fig. 7). The b-value fluctuated between 0.8 and 1.0 until early 2020, increased to 1.14 in mid-2020, and then decreased to about 0.75 in early 2023 and 0.72 by May 2025 (Fig. 7)^33^.

## Discussion

The sequences of unrest at Sierra Negra and Campi Flegrei show increasing rates of ground uplift and of VT seismicity with time—consistent, respectively, with an increase in applied loading and crustal fracturing. They superficially appear to show deformation and seismicity being driven by an increasing mean stress, *σ*_*d*_. Our analysis, however, shows that *σ*_*d*_ only increases during early unrest, when rates of seismicity are small, and that the significant increases in VT event rate and ground uplift coincide with a decrease in *σ*_*d*_ (Fig. 7).

The decrease in mean stress occurred even though the source pressure was increasing (Figs. 5 and 7). It is thus an internally-driven change and not the result of variations in external forcing. Rates of seismicity and ground movement can increase under smaller stresses only if the crust is becoming weaker. Weakening is an inevitable result of increased fracturing. A self-sustaining state can thus be recognised for which (1) fracturing reduces the crust’s strength and promotes faulting and (2) faulting releases stored energy and reduces the mean stress to a level that remains sufficient to overcome the crust’s smaller resistance, so that (3) additional fracturing occurs to repeat the cycle. Hence, in this framework, a decrease in mean stress provides a direct measure of progressive crustal weakening.

**Fig. 8:**
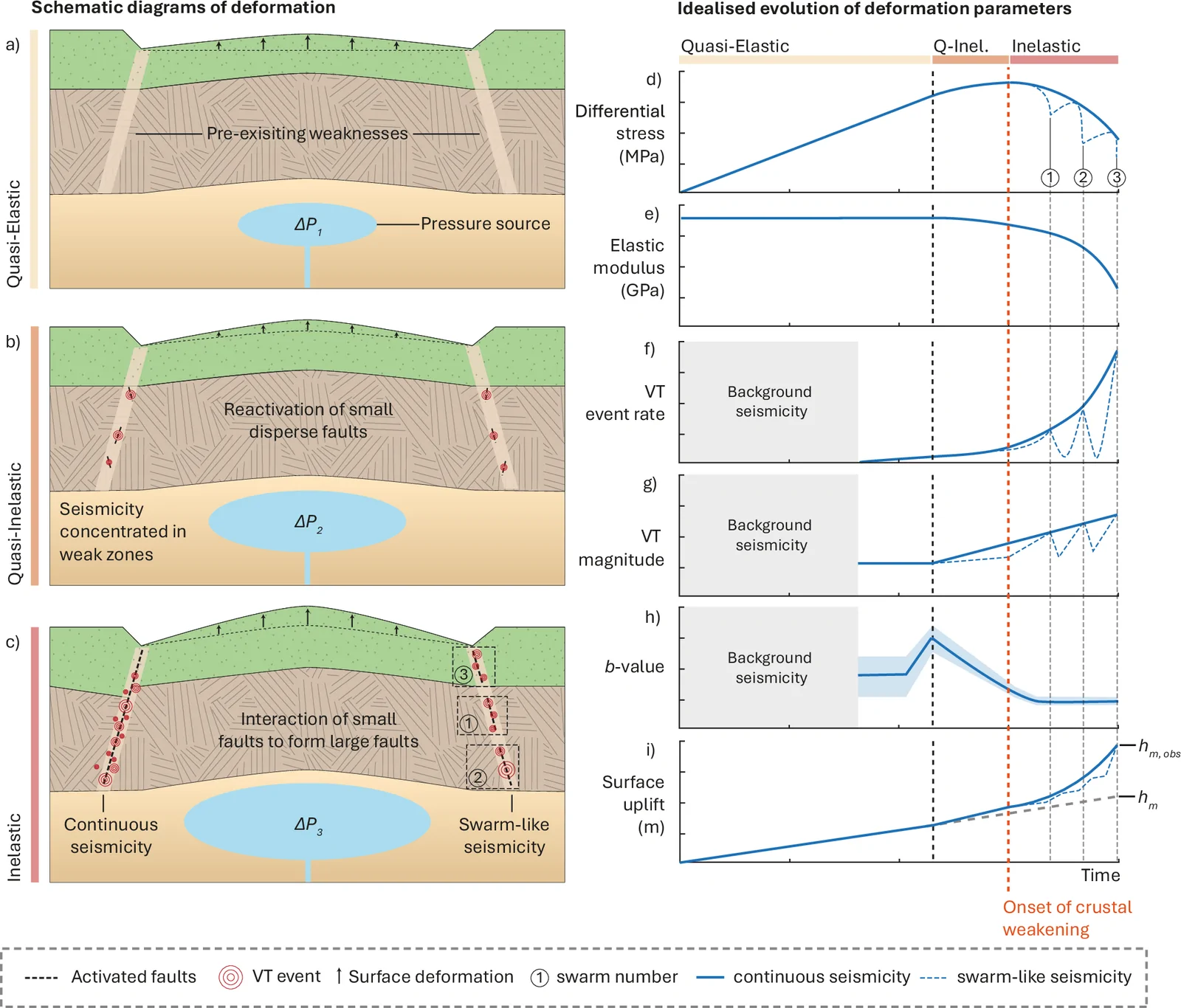
Conceptual evolution of fracturing and precursory signals across deformation regimes. **a**–**c** Schematic diagrams linking deformation regime to the inferred style of fracturing: **a** quasi-elastic deformation with minor fracturing; **b** quasi-inelastic deformation in which fracturing accelerates as diffuse, low-magnitude VT event; **c** inelastic deformation in which small-scale fractures interact and coalesce to form larger structures. Idealised evolution of precursory signals through time for each deformation phase: **d** differential stress, **e** elastic modulus, **f** VT event rate, **g** VT event magnitude, **h** seismic *b*-value, and **i** surface uplift. Solid curves show the expected evolution for continuous fracturing, whereas dashed curves indicate an alternative swarm-like evolution.

The transition from increasing to decreasing mean stress coincided with an evolution from approximately elastic to inelastic deformation. Incorporating the changes in uplift and seismicity rates (Fig. 8), the evolution can be subdivided into three stages. While the mean stress was increasing, behaviour evolved from (1) quasi-elastic (low rates of seismicity with uplift rates proportional to source pressurisation) to (2) quasi-inelastic deformation, in which fault movement contributed an increasing fraction of the total deformation and uplift rates exceed those of the quasi-elastic regime. This transitional stage is marked by inelastic processes that increasingly control deformation even as the mean stress continued to increase: the added inelastic strain accelerated uplift, so the uplift rate was no longer proportional to the pressurisation rate. Subsequently, when the mean stress began to decrease, deformation entered (3) inelastic behaviour, in which fault movement dominated and uplift remained faster than the quasi-elastic rate. Monitoring at Sierra Negra began during quasi-elastic deformation (2010–mid 2016), changed to quasi-inelastic in mid-2016, and became inelastic from late 2017 until the eruption on 26 June 2018. All three stages are observed at Campi Flegrei: quasi-elastic from the start of uplift in 2005 until 2020, quasi-inelastic from mid-2020 to late 2023, and inelastic since late 2023.

The evolution from quasi-elastic to inelastic behaviour also coincided with changes in the characteristics of the VT-event population. During the quasi-elastic phase, maximum VT magnitudes increased gradually, whereas b-values varied without a clear systematic trend (Fig. 7). Quasi-inelastic and inelastic behaviour were subsequently marked by continued increases in maximum magnitude, accompanied by a distinct and sustained decrease in *b*-value before it stabilised at a near-constant value (Fig. 7). The changes during the later stages indicate a progressive increase in both the maximum dimensions of slipping faults, reflected by the increasing earthquake magnitudes, and the relative proportion of larger events, reflected by the decreasing *b*-values^67^. The overall evolution was therefore consistent with the activation of a growing proportion of larger faults during the quasi-inelastic and inelastic regimes, together with enhanced fracture growth and linkage both between neighbouring faults and along developing detachment surfaces^8,68,69^. The changes in *b*-value also support the interpretation that smaller values are indicative of larger differential stresses^70^, but with the important caveat that this change typically occurred within a narrow window during the quasi-inelastic regime, when the mean stress approached its peak value. The sustained decrease in *b*-value occurred before the transition to inelastic deformation and, as a result, may be a potential precursor to the onset of crustal weakening by fracture^67^.

While both volcanoes followed this general evolution, the temporal expression of inelastic behaviour varied between them. At Sierra Negra, mean stress decreased steadily, with continuous uplift and seismicity, a pattern consistent with the gradual coalescence of small fractures into larger structures. Campi Flegrei, by contrast, exhibited stepwise decreases in the mean stress associated with discrete seismic swarms and periods of quiescence. Such an episodic style of fracturing is characteristic of failure in heterogeneous brittle materials^10^, and may reflect a greater amount of localised interaction of faults or spatial clustering of fracturing at Campi Flegrei (Fig. 8; see refs. ^16,71^). During the seismic swarms at Campi Flegrei, the uplift rate accelerated before returning to the rate associated with earlier quasi-elastic deformation. This behaviour supports the hypothesis that pressurisation occurs at a constant rate, and that transient increases in uplift are linked to elevated seismicity rates and crustal weakening. Notably, swarm-like seismicity occurred at Campi Flegrei, where the average strain rate is low (0.20 mm day^−1^), while continuous seismicity characterised Sierra Negra, where the strain rate is higher (0.82 mm day^−1^).

The evolution of earthquake characteristics and mean stress at both volcanoes is consistent with the behaviour observed during the tensile deformation of brittle materials in laboratory experiments. In particular, the changes observed during the inelastic regime mirror the strain-softening behaviour that typically characterises the final stages of deformation prior to bulk failure^16,71,72^. Differences in the style of inelastic deformation, ranging from continuous to swarm-like fracturing, are also consistent with experimental observations indicating that fracture style depends on strain rate: lower rates promote intermittent, swarm-like activity, whereas higher rates favour more continuous fracturing^71^.

Our conceptual framework highlights fracturing as a fundamental process governing the evolution of deformation at volcanoes and provides an explanation for observed changes in unrest prior to crustal rupture. Importantly, similar behaviour is observed in contrasting volcanic settings, despite differences in crustal structure and hydrothermal conditions. Both volcanoes exhibit the same characteristic transition from quasi-elastic to inelastic behaviour, suggesting that pressurisation-driven fracturing and progressive crustal weakening exert a generic first-order control on the evolution of unrest.

Although this behaviour may indicate the approach to crustal rupture, it does not necessarily constitute a precursor to eruption. For an eruption to occur, the rupture must be exploited by a fluid sufficiently pressurised to reach the surface. The probability that rupture will lead to an eruption thus remains an open question and lies beyond the scope of this study.

Additional processes may influence the elastic strain energy stored in the crust, potentially altering the progression of fracturing. They include variable rates of source pressurisation, tectonic earthquakes, hydrothermal activity and viscoelastic behaviour of the crust. For example, we have assumed that strain energy is transferred to the crust under an approximately constant rate of pressurisation. The rate of transfer will vary if the rate of pressurisation changes through time (e.g. owing to variations in the supply of pressurising fluid). However, as shown for Campi Flegrei in Supplementary Information C, although variable rates of pressurisation may alter the magnitude of stress changes in the crust, they are expected to have only a minor influence on when deformation changes from quasi-elastic to inelastic.

We similarly expect that the other processes will have a secondary influence relative to the long-term trends considered here. Thus: (1) large regional earthquakes may transiently perturb the background stress and energy state through dynamic shaking and step-like static offsets. However, such effects are likely to be short-lived relative to the sustained, pressurisation-driven trends that are the focus of our analysis. (2) Hydrothermal processes may modify effective stress by changing pore pressure and frictional strength: increasing pore pressure reduces effective normal stress and can promote fault slip and damage accumulation, whereas decreasing pore pressure increases effective normal stress and can inhibit slip. These effects can be accommodated within the present framework through an additional time-dependent term in the energy balance, without requiring a distinct deformation regime. The similar behaviour observed at Sierra Negra and Campi Flegrei supports this interpretation, despite the fact that sustained hydrothermal activity at Sierra Negra is limited and spatially restricted^23^, whereas Campi Flegrei hosts a vigorous hydrothermal system. (3) Country rock adjacent to a magma body may develop a high-temperature boundary layer that behaves viscoelastically; viscous deformation dissipates a fraction of the work done by the pressure source, reducing the energy stored elastically in the surrounding crust^73^. We account for this effect by introducing a dissipative term into Eq. (10) (Supplementary Information E), and find that although absolute stress magnitudes are sensitive to dissipation, the temporal evolution and timing of peak stress remain fixed.

By evaluating the elastic strain energy stored in the crust, it is possible to distinguish whether fracturing is driven by an increase in mean stress (quasi-elastic and quasi-inelastic regimes) or results from a decrease in the mean stress associated with progressive decrease in the bulk strength of the crust (inelastic regime). In the former, fracturing activates a distributed collection of existing faults; in the latter, it reflects the coalescence of fractures into larger structures, increasing crustal damage and potentially forming a connected pathway between the pressure source and the surface. The distinction is important for interpreting unrest. For example, an acceleration in seismicity during the quasi-elastic period poses a lower risk than a similar change during the inelastic period.

Our results present a practical method for evaluating temporal changes in the mean stress within the volcanic crust during periods of unrest.They show that mean stress increases until failure is approached and then decreases, consistent with a progression from quasi-elastic, through quasi-inelastic, to inelastic behaviour. This evolution implies that interpretations made during the early stages of unrest, when stress is increasing, cannot be assumed to remain valid once the crust enters a weakening regime and mean stress begins to decrease. In particular, accelerations in VT seismicity do not necessarily indicate a further increase in differential stress. Instead, at later stages, they may signal the growth and coalescence of major fractures in a weakening crust before failure. An increase in VT event rate under decreasing mean stress is thus a crucial additional criterion for recognising the approach to crustal rupture and a possible eruption.

## Methods

### Elastic strain energy and mean stress in the crust

The change in mean differential stress in volcanic crust during unrest is determined by the resultant elastic strain energy stored in the crust. This represents the change in elastic strain energy relative to the start of the analysis period and is defined in excess of any pre-existing background strain energy. The resultant energy ($\overline{U}$) reflects a balance between elastic strain energy transferred to the crust by an expanding pressure source (*U*_*o*_), elastic strain energy released by seismicity (*U*_*s*_), and any contribution from regional tectonic loading (*U*_*r*_), such that

4 $$\overline{U}={U}_{o}+{U}_{r}-{U}_{s}.$$

In this study, we consider a pressure source of arbitrary shape confined in a homogeneous, isotropic elastic medium in which regional tectonic deformation is negligible, so that *U*_*r*_ = 0. This assumption is reasonable for both case studies. At Campi Flegrei, the regional stress field has been shown to be minor compared to volcanic forcing during unrest episodes^49^. Sierra Negra is located within the interior of the northern Nazca Plate^20^. Residual GPS velocities indicate that internal deformation of the plate occurs at a rate approximately three orders of magnitude lower than the local deformation rates associated with volcanic unrest^74^. Therefore, over the timescale of unrest and the spatial scale of the volcanic edifice, the strain associated with regional tectonic deformation is negligible relative to volcanically induced strain. Consequently, the regional loading term *U*_*r*_ is omitted for both case studies. Regional tectonic deformation could nevertheless be incorporated into the framework by using internally generated strain rates associated with regional loading to estimate the additional elastic strain energy accumulated in the crust^50,75^.

We interpret the observed deformation in terms of net pressurisation of the source; processes such as permeability change of the crust and degassing are not modelled explicitly. The source has an internal pressure, *P*, in excess of the lithostatic pressure. As the internal pressure increases, the source expands and deforms the surrounding crust. The work for deformation is given by the product of the internal pressure and the change in volume of the source, *ΔV*, both of which are functions of time^50,72^. The work is stored as elastic strain energy, *U*_*o*_, given by ref. ^50^

5 $${U}_{o}(t)=\frac{1}{2}P(t)\Delta V(t).$$

Here, Δ*V* denotes the change in volume of the deforming source (i.e. the cavity/reservoir), not the change in volume of the pressurising fluid. In the elastic approximation, *P* and Δ*V* are linked by the source compliance; Eq. (5) therefore represents the work done by net pressurisation. In practice, crustal layering and mechanical heterogeneity primarily modify the effective elastic stiffness governing the deformation-pressure relationship; we therefore treat the shear modulus *G* as an effective bulk parameter and propagate plausible uncertainty in *G* through the energy and stress estimates (Supplementary Information D).

The elastic strain energy stored in the surrounding crust is partitioned into volumetric and distortional components^72^. The volumetric component represents elastic compression or dilation of the crust under hydrostatic stress. The distortional component represents shear-related deformation associated with changes in shape at approximately constant volume and is directly linked to deviatoric stress, fault slip, and brittle failure. Since volcanic deformation is dominated by shape change of the crust rather than bulk volumetric changes^2^, we assume that the energy transferred to the crust by source pressurisation is predominantly stored as distortional (shear-related) elastic strain energy. This assumption is quantitatively supported by the spatial partitioning of elastic strain energy for sill-shaped inflation, which shows that the distortional component accounts for approximately 81% and 92% of the total elastic strain energy for representative source geometries at Sierra Negra and Campi Flegrei, respectively (Supplementary Information B).

Prior to the onset of significant seismicity, the movement of the crust is predominantly elastic and the surface deformation is proportional to the internal pressure of the source. As pressurisation continues the differential stress in the crust approaches the elastic limit, and a significant component of deformation is accommodated inelastically, by fault movements that trigger VT seismicity^8^. Each VT earthquake releases an amount Δ*U*_*e*_ of the stored elastic strain energy, partitioned into radiated seismic energy, *E*_*r*_ and non-radiated energy, *E*_*n*_, dissipated by processes such as frictional heating and the creation of new fracture surfaces^76–78^, so that

6 $$\Delta {U}_{e}={E}_{r}+{E}_{n}.$$

We estimate *E*_*r*_ in Joules from the moment magnitude using the empirical magnitude-energy scaling^51^

7 $${\log }_{10}\left({E}_{r}\right)=1.5{M}_{w}+4.8,$$

The fraction of the elastic strain energy radiated as seismic energy (*E*_*r*_/Δ*U*_*e*_) is the seismic efficiency, *η*,^78,79^. For small magnitude earthquakes in the shallow crust (*M*_*w*_ < 3.3), published estimates indicate typical values of order 10^−2^, but with substantial event-to-event variability spanning more than one order of magnitude^66^. Accordingly, we estimate the elastic strain energy released by an individual VT earthquake as

8 $$\Delta {U}_{e,i}=\frac{{E}_{r,i}}{{\eta }_{i}}.$$

Rather than prescribing a single value of *η*, we account for this variability by allowing *η* to vary between events and by propagating its uncertainty through the model using a Monte Carlo approach (Supplementary Information D). The cumulative elastic strain energy released by seismicity is therefore given by

9 $${U}_{s}(t)={\sum}_{i=1}^{N}\Delta {U}_{e,i}({t}_{i}),$$

where *N* is the number of VT earthquakes that have occurred up to time *t*. In this framework, earthquakes are treated as an energy sink that reduces the elastic strain energy stored in the crust. We do not consider dynamic stress changes associated with the transmission of seismic waves, because they operate on timescales much shorter than those resolved by our analysis. The elastic strain energy released by VT earthquakes is expected to be predominantly distortional, as the vast majority of events at both Campi Flegrei and Sierra Negra display focal mechanisms indicative of shear failure on faults rather than volumetric sources such as explosions or implosions^21,80^. Consequently, both the energy transferred to the crust by pressurisation and the energy released by seismicity are treated as distortional elastic strain energy in the following analysis.

The time evolution of the elastic strain energy stored in the crust, $\overline{U}(t)$, is given by

10 $$\overline{U}(t)=\alpha {U}_{o}(t)-{\sum }_{i=1}^{N}\Delta {U}_{e,i}({t}_{i}),$$

where *U*_*o*_(*t*) is the distortional elastic strain energy transferred to the crust by the pressure source, *α* is a dimensionless factor representing the fraction of source work stored as distortional elastic strain energy in the crust, and the summation term is the cumulative distortional elastic strain energy released by VT earthquakes. In the case *α* = 1 all work done by the pressurising source is transferred to distortional elastic strain energy, whereas *α* < 1 allows for dissipation (e.g. viscoelastic relaxation). For simplicity we set *α* = 1 in the main analysis; the implications of relaxing this assumption are explored in Supplementary Information E.

The stored energy increases when $\overline{U} > 0$, decreases when $\overline{U} < 0$ and remains unchanged when $\overline{U}=0$. We assume that the stored energy is homogeneously distributed throughout a characteristic volume *V*_*d*_ of the crust.

Given that both the energy transferred to the crust and the energy released by seismicity are primarily distortional, we equate the stored elastic strain energy (Eq. (10)) with the definition of distortional elastic strain energy^72^, yielding

11 $$\frac{\overline{U}}{{V}_{d}}=\frac{1}{12G}\left[{({\sigma }_{1}-{\sigma }_{2})}^{2}+{({\sigma }_{2}-{\sigma }_{3})}^{2}+{({\sigma }_{3}-{\sigma }_{1})}^{2}\right],$$

where *G* is the shear modulus of the material and *σ*_1_, *σ*_2_, *σ*_3_ are the principal components of stress. Eq. (11) connects the elastic strain energy to the differential stresses, equivalent in magnitude to the deviatoric stress, represented by the terms (*σ*_*i*_ − *σ*_*j*_) on the right-hand side. The square root of ${({\sigma }_{1}-{\sigma }_{2})}^{2}+{({\sigma }_{2}-{\sigma }_{3})}^{2}+{({\sigma }_{3}-{\sigma }_{1})}^{2}$ provides a scalar measure of the magnitude of differential stress and does not depend on the coordinate system used. As such we introduce *σ*_*d*_ as a measure of the differential stress in the system, where

12 $${\sigma }_{d}=\frac{1}{\sqrt{2}}{\left[{({\sigma }_{1}-{\sigma }_{2})}^{2}+{({\sigma }_{2}-{\sigma }_{3})}^{2}+{({\sigma }_{3}-{\sigma }_{1})}^{2}\right]}^{\frac{1}{2}}.$$

Failure occurs when *σ*_*d*_ exceeds a critical value. For shear failure, the critical stress has been adopted as a yield criterion based on the concept of maximum distortion energy^52^. Here, we use *σ*_*d*_ to identify the differential stress at which brittle failure begins. For example when any two principal stresses are equal, (*σ*_1_ = *σ*_2_ or *σ*_2_ = *σ*_3_), Eq. (12) simplifies to the maximum differential stress *σ*_*d*_ = (*σ*_1_ − *σ*_3_), which is equal to twice the maximum shear stress.

Substituting Eq. (12) into Eq. (11), the representative differential stress is given by

13 $${\sigma }_{d}(t)=\sqrt{6G\frac{\overline{U}(t)}{{V}_{d}}}.$$

Here, *σ*_*d*_ is used as a consistent and systematic measure of relative changes in differential stress and is not used determine absolute stress magnitudes.

### Estimation of *b*-value

We estimate the Gutenberg-Richter *b*-value using the maximum likelihood method^12,81^, given by

14 $$b=\frac{1}{\ln (10)(\overline{M}-{M}_{c})}$$

where $\bar{M}$ is the mean magnitude and *M*_*c*_ is the completeness magnitude of the seismic catalogue. To assess temporal variations in the *b*-value, we apply this method within a 6-month moving window, considering only events above *M*_*c*_ and ensuring a minimum of 50 events in each subset^82^. The associated uncertainty is estimated using the standard deviation of the *b*-value^82^, defined as

15 $${\sigma }_{b}=2.30{b}^{2}\sqrt{\frac{\mathop{\sum }_{i=1}^{N}{({M}_{i}-\overline{M})}^{2}}{N(N-1)}},$$

where *N* is the number of earthquakes in the window.

## Supplementary information

Supplementary Information

Transparent Peer Review file

## Data Availability

For Campi Flegrei, the seismic data were obtained from https://doi.org/10.13127/gossip^32^ and the uplift data from^83^ (2000-2024) and the monthly bulletins of the INGV-Osservatorio Vesuviano (2024-2026). For Sierra Negra, the seismic and uplift data were obtained from ref. ^21^.
