# Supplementary material for: Crustal weakening before rupture at volcanoes after long repose
Source: Nat Commun. 2026 Aug 24;17:10152. doi: 10.1038/s41467-026-77001-5 (PMC13601623; doi:10.1038/s41467-026-77001-5)
Supplement: Supplementary file 1 [file 41467_2026_77001_MOESM1_ESM.pdf]

Supplementary Information

to

Crustal weakening before rupture at volcanoes after long repose

Eric L. Newland[1*] & Christopher R. J. Kilburn [1]

[1] UCL Hazard Centre, Department of Earth Sciences, University College London, London, United Kingdom

[*] email: px26843@bristol.ac.uk

This PDF file includes:

Supplementary Methods

Section A: Determination of quasi-elastic transition date

Supplementary Discussion

Section B: Elastic strain-energy budget

Section C: Sensitivity to uplift parameterisation at Campi Flegrei

Section D: Uncertainty in energy and stress estimates

Section E: Viscoelastic relaxation

Supplementary References

A: Determination of quasi-elastic transition date

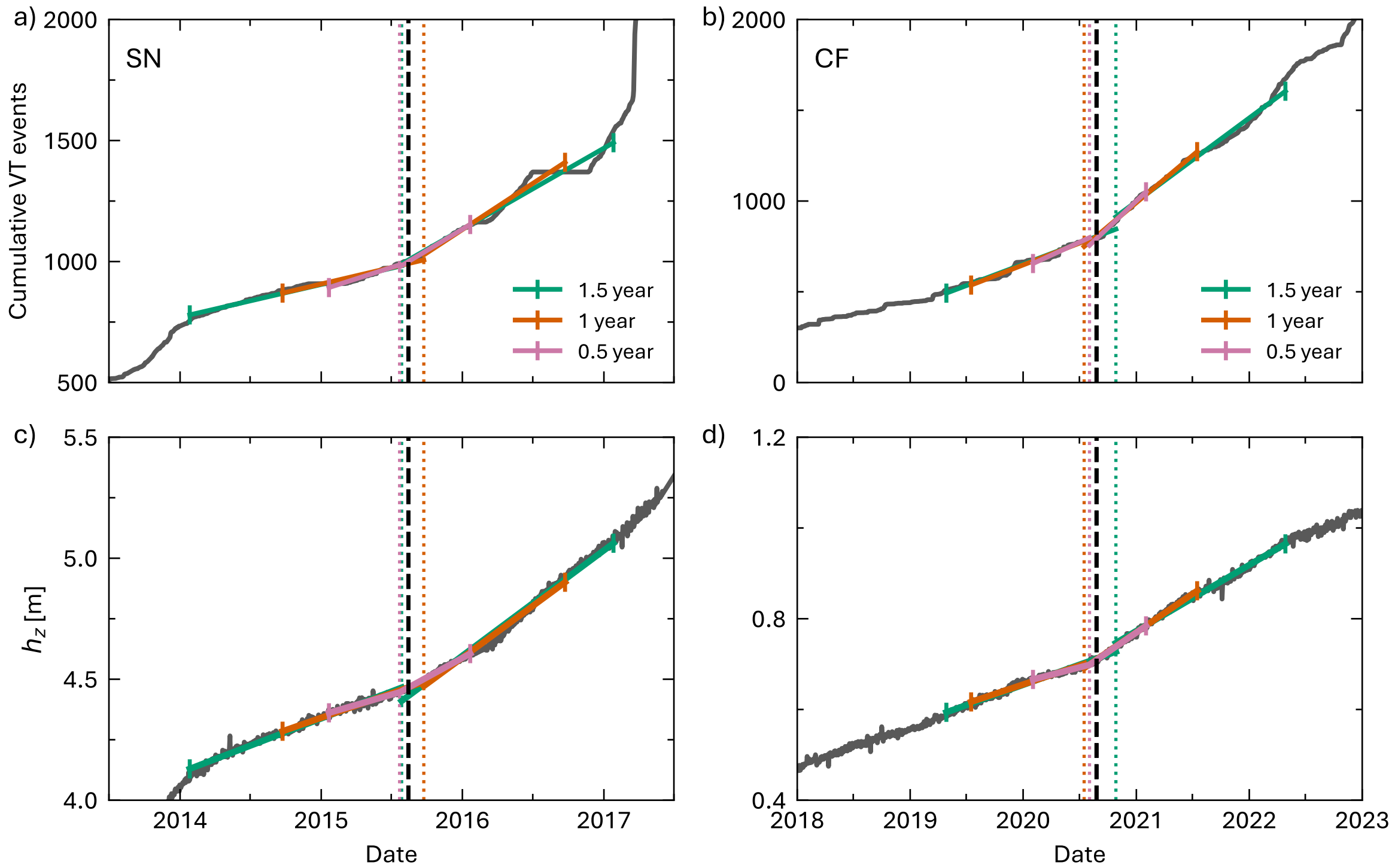

Figure S1: Sensitivity of the inferred transition dates to the length of the sliding window. Cumulative VT event counts at (a) Sierra Negra and (b) Campi Flegrei, and cumulative vertical uplift at (c) Sierra Negra and (d) Campi Flegrei. Coloured lines show the linear fits immediately before and after the inferred transition using window lengths of 0.5, 1, and 1.5 years. Corresponding vertical lines show the inferred transition dates.

We define the end of the quasi-elastic regime as the point at which the uplift rate begins to increase above its previously near-constant value, coincident with an increase in the VT seismicity rate. Because these changes are difficult to identify directly from the event-rate time series, we estimate the transition dates from the cumulative number of VT events and the cumulative vertical uplift. The gradients of these records correspond to the VT event rate and uplift rate, respectively, allowing sustained changes in rate to be identified more clearly.

For each candidate transition date, we fit linear regressions to equal-duration windows immediately before and after that date. The change in rate is quantified as the difference between the fitted gradients, normalised by their combined standard errors. The analysis is applied separately to the cumulative VT event and uplift records at Sierra Negra and Campi Flegrei. For each volcano, the transition date is defined by the joint maximum in the normalised gradient changes of the two records, identifying the onset of a sustained increase in both uplift and seismicity rates. Using this approach, we identify transition dates of 14 August 2015 at Sierra Negra and 25 August 2020 at Campi Flegrei.

To assess the sensitivity of the inferred transition dates to the chosen window length, we repeat the analysis using windows of 0.5, 1, and 1.5 years (Fig. S1). Although the fitted gradients and the magnitude of the calculated rate changes vary with window length, the inferred transition dates remain closely grouped.

B: Elastic strain-energy budget

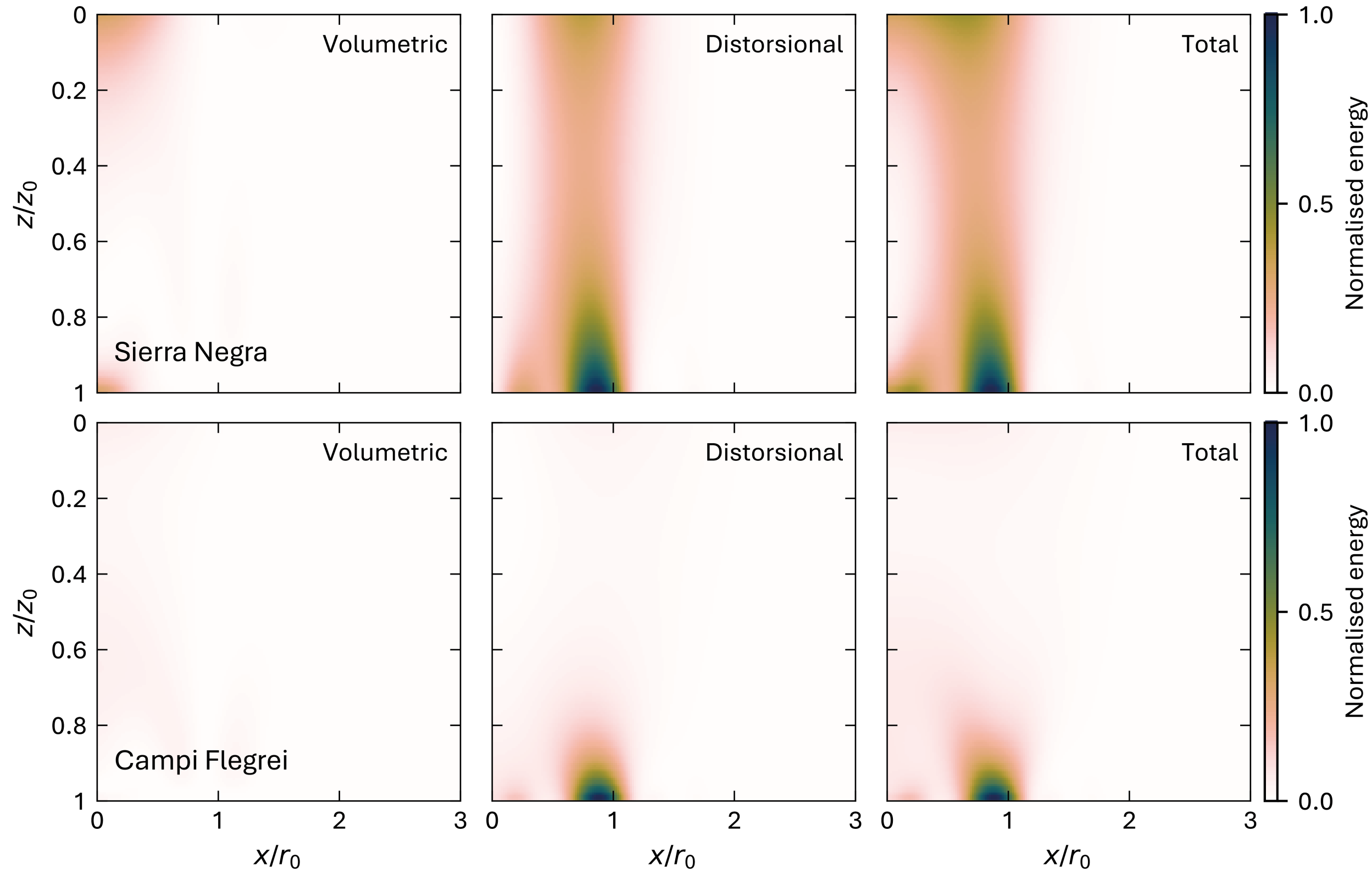

Figure S2: Radial cross sections showing the volumetric (left), distortional (centre), and total (right) elastic strain-energy density for Sierra Negra (top row) and Campi Flegrei (bottom row), calculated using the penny-shaped crack model. Energies are normalised by the maximum total strain energy in each case. Coordinates are shown in terms of horizontal distance, x/r_0, and depth, z/z_0, relative to the source radius and depth.

To constrain the partitioning and spatial distribution of elastic strain energy in the crust resulting from the expansion of a sill-like source, we use the penny-shaped crack model of [1], as implemented by [2]. Internal strain due to sill-like inflation is calculated from the components of the displacement gradient tensor, obtained using centred finite-difference derivatives (second-order accurate). The infinitesimal strain tensor at each coordinate is given by the symmetric part of the displacement gradient tensor,

$$\boldsymbol{\varepsilon} = \tfrac{1}{2}\left(\nabla \mathbf{h} + (\nabla \mathbf{h})^{\mathsf{T}}\right), \tag{S1}$$

or, in component form,

$$\varepsilon_{ij} = \frac{1}{2}\left(\frac{\partial h_i}{\partial x_j} + \frac{\partial h_j}{\partial x_i}\right), \tag{S2}$$

where $\mathbf{h} = (h_x, h_y, h_z)$ is the displacement vector and (x, y, z) are Cartesian coordinates (with $i, j \in x, y, z$). Each displacement-gradient component is approximated using centred finite differences, e.g.

$$\frac{\partial h_x}{\partial x} \approx \frac{h_x(x+\Delta x, y, z) - h_x(x-\Delta x, y, z)}{2\Delta x}, \tag{S3}$$

$$\frac{\partial h_x}{\partial y} \approx \frac{h_x(x, y+\Delta y, z) - h_x(x, y-\Delta y, z)}{2\Delta y}, \tag{S4}$$

with analogous expressions for derivatives with respect to z and for h_y and h_z.

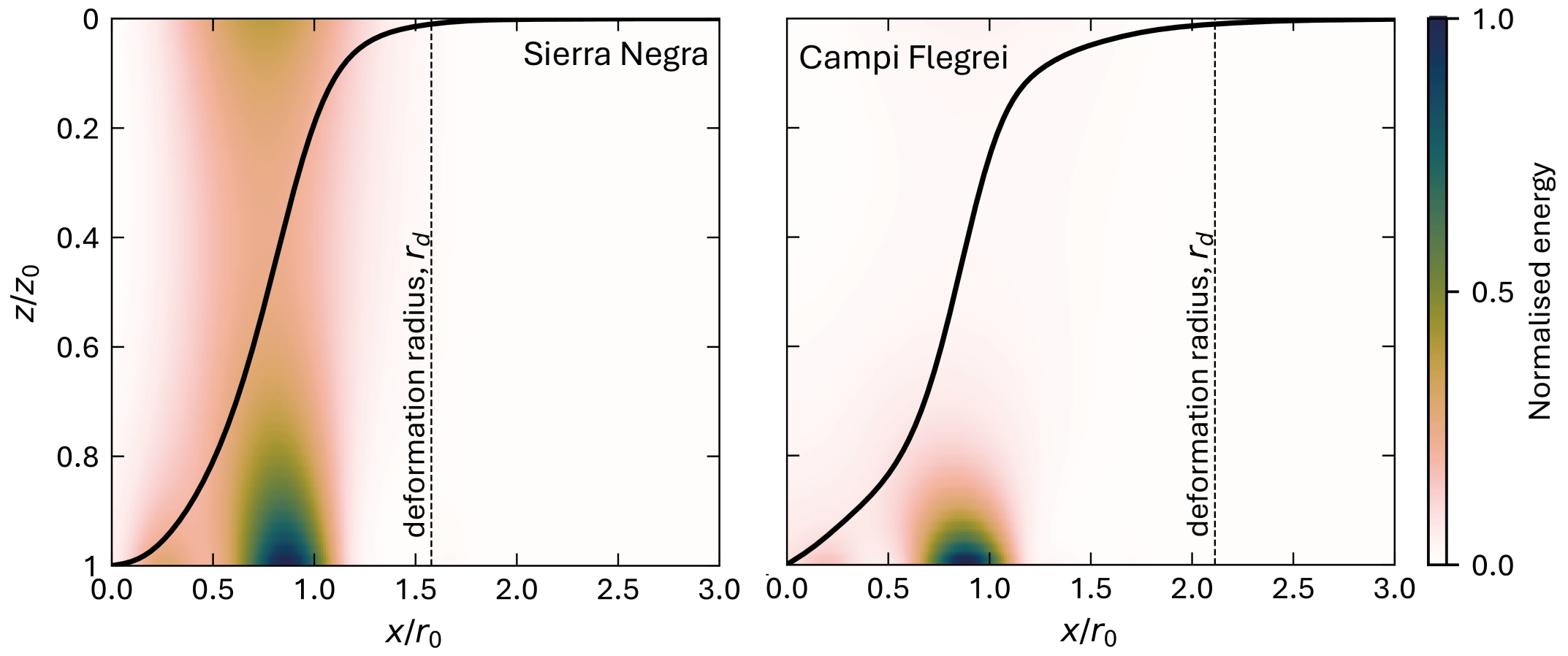

Figure S3: Radial cross sections of normalised deviatoric elastic strain-energy density (colour scale) and cumulative radial integration of deviatoric strain energy (solid black line) for Sierra Negra (left) and Campi Flegrei (right). The cumulative energy is obtained by integrating the deviatoric strain-energy density outward from the source centre. The dashed vertical line marks the deformation radius, r_d, defined as the radial distance enclosing 99% of the total deviatoric elastic strain energy.

We then use the strain tensor to compute the volumetric (dilatational) and deviatoric (distortional) contributions to the elastic strain-energy density. The strain is first decomposed into its volumetric and deviatoric parts,

$$\varepsilon_m = \tfrac{1}{3}\varepsilon_{kk}, \qquad \varepsilon'_{ij} = \varepsilon_{ij} - \varepsilon_m \delta_{ij}, \tag{S5}$$

where $\varepsilon_{kk} = \varepsilon_{xx} + \varepsilon_{yy} + \varepsilon_{zz}$ is the trace of the strain tensor and δ_{ij} is the Kronecker delta. Assuming linear isotropic elasticity, the elastic strain-energy density is

$$U = \tfrac{1}{2}\sigma_{ij}\varepsilon_{ij} = U_{\text{vol}} + U_{\text{dev}}, \tag{S6}$$

with volumetric and deviatoric components given by

$$U_{\text{vol}} = \tfrac{1}{2}K\left(\varepsilon_{kk}\right)^2, \tag{S7}$$

$$U_{\text{dev}} = G\varepsilon'_{ij}\varepsilon'_{ij}, \tag{S8}$$

where K is the bulk modulus and G is the shear modulus.

The partitioning of elastic strain energy is obtained by evaluating U_{vol} and U_{dev} at each point in the model domain. Here, the volumetric component reflects elastic compression or dilation of the surrounding crust, not volume changes of magma or pore fluids within the source. The distortional component represents shear-related deformation associated with changes in shape at approximately constant volume, and is therefore the component directly relevant to fault slip and brittle failure. Fig. S2 shows the resulting distributions in a radial cross section of volumetric, distortional, and total elastic strain energy, normalised by the maximum total energy. The volumetric component is spatially diffuse and contributes weakly to the total energy budget, whereas the distortional component is strongly localised above the sill-like source and dominates the total elastic strain energy. As a result, the spatial pattern of total strain energy closely follows that of the deviatoric contribution, indicating that deformation associated with sill-like inflation is primarily accommodated by shear rather than volumetric dilation. Integrating the volumetric and deviatoric contributions over the full model domain for the source conditions inferred at Sierra Negra and Campi Flegrei shows that the deviatoric strain energy represents 81% and 92% of the total strain energy, respectively. These results demonstrate that, for sill-like sources and elastic parameters appropriate to Sierra Negra and Campi Flegrei, the mechanically relevant elastic strain energy stored in the crust is

dominated by its distortional component, justifying the assumption adopted in the main text.

To quantify the spatial extent of deformation, the deviatoric strain-energy density is radially integrated outward from the source centre to obtain the cumulative energy as a function of distance (Fig. S3). The cumulative energy increases rapidly near the source and asymptotically approaches the total stored energy. We define the deformation radius, r_d, as the radial distance enclosing 99% of the deviatoric elastic strain energy. For the source properties inferred at Campi Flegrei we obtain $r_d = 2.10, r_0$, whereas for Sierra Negra we obtain $r_d = 1.60, r_0$. This provides an objective measure of the crustal volume that is mechanically involved in accommodating pressurisation-induced deformation. Notably, r_d extends beyond the physical radius of the sill-like source, demonstrating that the mechanical influence of sill inflation is distributed over a substantially larger region of the surrounding crust.

Integrating the deviatoric strain-energy density within the deformation volume V_d yields an independent estimate of the deviatoric strain energy stored in the crust. For a nominal overpressure of 10 MPa, the source-work estimate from Eq. 10 (Methods) gives 1.27×10^{14} J for Campi Flegrei, whereas full spatial integration yields 1.00×10^{14} J. For Sierra Negra, the corresponding values are 2.74×10^{14} J (Methods; Eq. 10) and 7.74×10^{14} J (full integration). Despite being derived from independent formulations, these values are of the same order and therefore provide a check on the energy calculation and the assumption that the work done by the pressurising source is predominantly stored as elastic strain energy.

C: Sensitivity to uplift parameterisation at Campi Flegrei

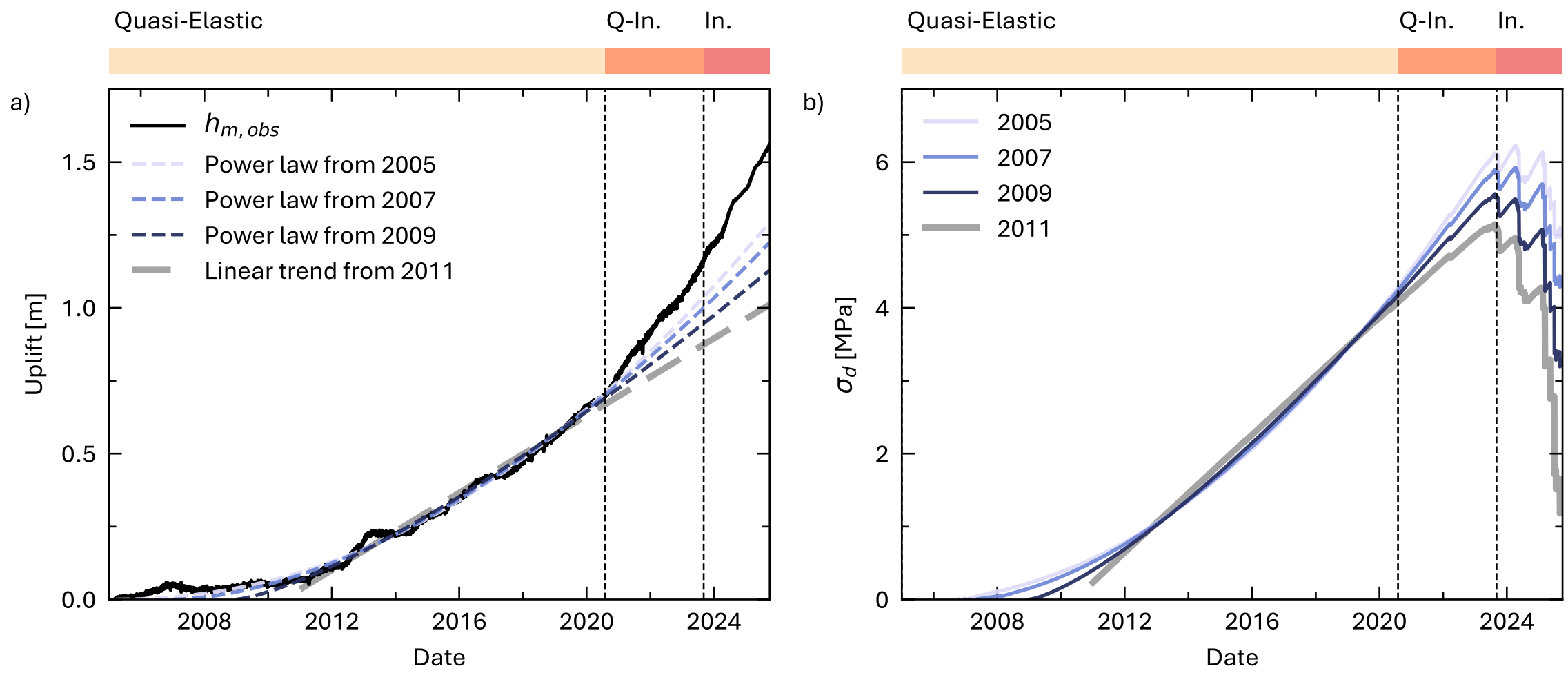

Figure S4: (a) Observed vertical uplift, $h_{m,\text{obs}}$, compared with alternative parameterisation of the quasi-elastic uplift history, including power-law fits initiated in 2005, 2007, and 2009, and the limiting linear trend fitted from 2011 and adopted in the main text. Shaded bars indicate the quasi-elastic, quasi-inelastic, and inelastic deformation regimes.(b) Corresponding evolution of mean differential stress, σ_d, obtained by propagating each uplift parameterisation through the same source-pressure inversion and energy-balance framework.

To assess the non-uniqueness of the inferred pressurisation rate and its impact on the evolution of mean stress at Campi Flegrei, we test the sensitivity of the results to both the choice of uplift parameterisation and the start date of the quasi-elastic fitting window.

The quasi-elastic regime is defined as the interval during which VT seismicity rates are low and deformation is dominated by elastic behaviour, such that the rate of surface uplift is proportional to the rate of source pressurisation. In the main text, we assume that both volcanoes are characterised by approximately constant pressurisation rates during this phase. At Sierra Negra, this assumption is well supported by the data, as uplift rapidly adjusts to a near-constant rate following an initial deceleration. At Campi Flegrei, however, uplift resumes after 20 years of subsidence and initially increases gradually before transitioning to a near-linear trend. As a result, the early adjustment phase and subsequent linear uplift could alternatively be interpreted as a single continuously accelerating trend rather than an adjustment followed by constant-rate pressurisation. This section addresses this non-uniqueness for Campi Flegrei.

The end of the quasi-elastic regime is held fixed in all tests, as it is independently determined from a joint change-point analysis of the uplift and VT seismicity time series, which identifies a contemporaneous acceleration in both signals in early 2020. To assess the impact of non-linear pressurisation histories prior to this transition, we fit the quasi-elastic uplift history using power-law functions initiated in 2005, 2007, and 2009, and compare these with the limiting linear trend fitted from 2011 and adopted in the main text (Fig. S4.a). Each fitted uplift history is then propagated through the same source-pressure inversion and energy-balance framework to compute the corresponding evolution of mean stress (Fig. S4.b).

Varying the start date and functional form of the uplift fit primarily affects the absolute magnitude of the inferred stress and stored elastic strain energy. In contrast, the temporal evolution of stress is robust: all tested parameterisations exhibit a progressive stress increase during the quasi-elastic phase, followed by a consistent timing of peak stress and a subsequent decline associated with enhanced energy loss. The inferred transitions between quasi-elastic, quasi-inelastic, and inelastic deformation regimes remain effectively unchanged across all tested scenarios.

These results demonstrate that, although the pressurisation history at Campi Flegrei is not uniquely

constrained by surface deformation alone, the identification and timing of deformation regime transitions are insensitive to the assumed uplift parameterisation and fitting window. The conclusions drawn in the main text therefore reflect robust features of the coupled deformation–seismicity evolution, rather than artefacts of a particular modelling choice.

D: Uncertainty in Energy and Stress Estimates

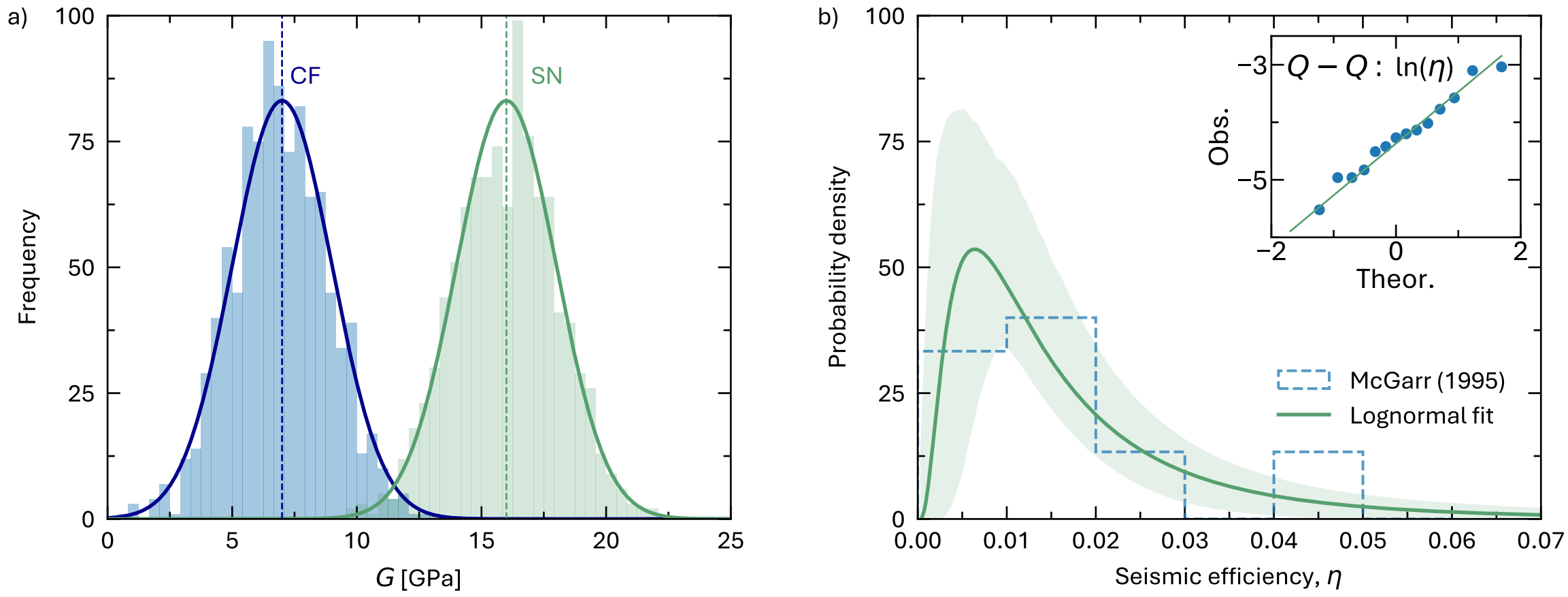

Figure S5: Parameter distributions used in the Monte Carlo uncertainty analysis. (a) Normal distributions of shear modulus, G, for Sierra Negra (teal) and Campi Flegrei (blue), with means of 16 GPa and 7 GPa, respectively, and standard deviation $\sigma_G = 2$ GPa (a single draw of G is used per model realisation). (b) Log-normal distribution of seismic efficiency, η. The distribution is fitted to the compilation of η values reported by [3] in log space, $\ln(\eta) \sim \mathcal{N}(\mu, \sigma^2)$; normality of $\ln(\eta)$ is supported by a Q–Q plot and a Shapiro–Wilk test ($p = 0.897$). The shaded region shows the confidence envelope of the fitted lognormal probability density obtained via a non-parametric bootstrap of the McGarr dataset (resampling $\ln(\eta)$ and re-estimating μ and σ by maximum likelihood).

In this section, we evaluate the sensitivity of the inferred elastic strain energy and stress evolution to uncertainties in (i) the elastic properties of the crust and (ii) the seismic efficiency used to convert seismic energy release into elastic strain energy loss. These two parameters represent the dominant sources of uncertainty in the energy-balance framework used in this study. To propagate these uncertainties through the model we use a Monte Carlo approach.

Uncertainty in elastic properties is represented by variability in the shear modulus, G. For each volcano, we assume that G is normally distributed, with a mean of 16 GPa for Sierra Negra and 7 GPa for Campi Flegrei, and a standard deviation of 2 GPa in both cases (Fig. S5.a). These distributions reflect plausible ranges for shallow volcanic crust and also represent uncertainty associated with approximating a layered and mechanically heterogeneous crust by a homogeneous elastic medium (i.e., G is treated as an effective bulk stiffness). For each model realisation, a single value of G is drawn from the appropriate distribution and applied consistently throughout the calculation.

Uncertainty in the fraction of elastic strain energy released seismically is captured through the seismic efficiency, η. Rather than prescribing a single value, we assign each earthquake in the seismic catalogue a seismic efficiency drawn independently from a predetermined distribution. Because η is strictly positive and spans more than one order of magnitude, we model event-to-event variability using a log-normal distribution, $\eta \sim \mathrm{LogNormal}(\mu, \sigma^2)$, equivalently $\ln(\eta) \sim \mathcal{N}(\mu, \sigma^2)$. We justify the log-normal form using the published compilation of η values for small-magnitude earthquakes reported by [3]. Normality of $\ln(\eta)$ is assessed using both a quantile-quantile plot and a Shapiro-Wilk test, which yields no evidence against normality ($p = 0.897$). Given the limited sample size ($n = 15$), the log-normal distribution provides a parsimonious and physically reasonable working model for η.

Because the fitted parameters (μ, σ) are uncertain owing to the small number of available measurements, we quantify this uncertainty using a non-parametric bootstrap. Working in log space, $y_i = \ln(\eta_i)$, we generate bootstrap datasets by resampling with replacement from the observed y_i values. For each bootstrap sample, maximum-likelihood estimates of μ and σ are computed. The resulting ensemble of (μ, σ) pairs defines an empirical distribution of plausible lognormal parameters consistent with the

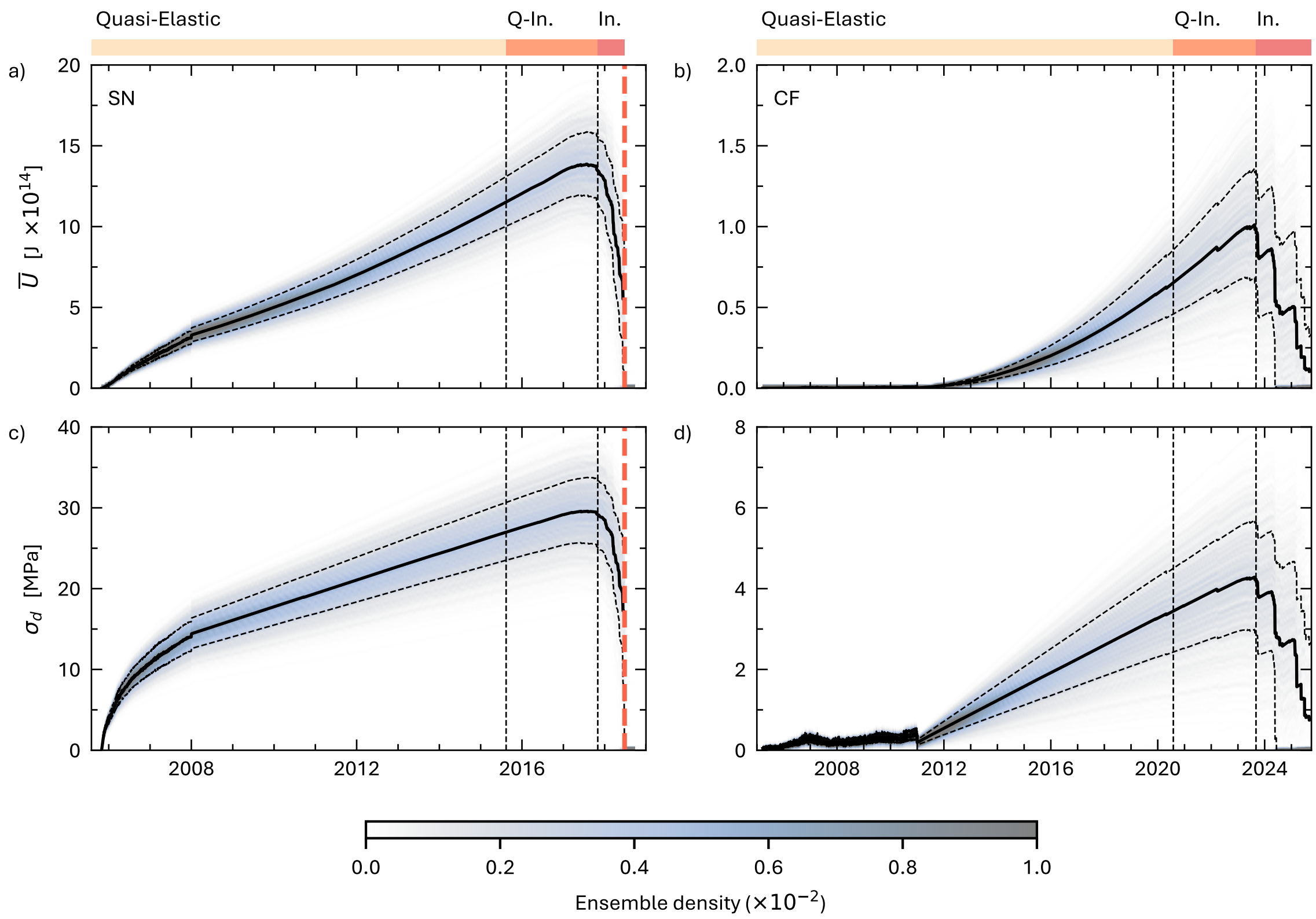

Figure S6: (a, c) Sierra Negra (2005–2019) and (b, d) Campi Flegrei (2011–2025-10). (a, b) Mean elastic strain energy, $\overline{U}$, stored in the crust through time. (c, d) Mean differential stress, σ_d, through time. Greyscale shading shows the density of values from 1000 Monte Carlo realisations of the stress-evolution model; the solid black line shows the ensemble mean and dashed black lines indicate ± 1 standard deviation. Vertical dashed lines mark the end of the quasi-elastic period and the transition to inelastic behaviour; the red dashed line marks the eruption date at Sierra Negra. Beige, orange and red bars indicate the quasi-elastic, quasi-inelastic and inelastic regimes, respectively.

available data and is visualised as a confidence envelope for the lognormal probability density function (Fig. S5.b). This procedure captures epistemic uncertainty in the distribution parameters arising from limited observations.

Uncertainty in G and η is propagated through the full stress-evolution model using a Monte Carlo approach. For each model realisation:

1. A value of G is drawn from its prescribed normal distribution.
2. A pair (μ, σ) is sampled from the bootstrap distribution.
3. Each earthquake in the seismic catalogue is assigned an independent seismic efficiency, $\eta_k \sim \mathrm{LogNormal}(\mu, \sigma^2)$.
4. The elastic strain energy released per event is calculated using the sampled η_k, and the cumulative energy balance and stress evolution are recomputed.

This procedure captures both event-to-event variability in η and uncertainty in the underlying distribution inferred from sparse data.

Using this framework, we run the full stress-evolution model 1000 times. At each time step, we compute the ensemble mean and standard deviation of elastic strain energy and mean stress. Figure S6 shows the resulting mean energy and stress evolution together with their associated uncertainty envelopes for Sierra Negra and Campi Flegrei.

Although uncertainties in elastic properties and seismic efficiency affect the absolute magnitudes of inferred elastic strain energy and stress, the overall temporal evolution is robust. Across the ensemble, the transition from quasi-elastic to inelastic behaviour occurs at consistent times, and the relative changes in stress through time are preserved. These results show that the principal conclusions of the study do not depend sensitively on the assumed values of G or η, but instead reflect robust features of the energy balance and seismicity during unrest. On this basis, we present only the ensemble mean and one standard deviation of elastic strain energy and mean stress in the main text, as these quantities adequately represent uncertainty while preserving the key temporal patterns identified in the analysis.

These results demonstrate that, although uncertainties in elastic properties and seismic efficiency affect the absolute scaling of inferred stress and energy, they do not alter the principal temporal patterns or transition points identified in the main analysis. The conclusions drawn from the stress-evolution framework are therefore robust to plausible uncertainties in these key parameters. On this basis, we present only the ensemble mean and one standard deviation of the inferred elastic strain energy and stress evolution in the main text, as these quantities adequately capture the uncertainty while preserving the robust temporal trends identified in the analysis.

E: Viscoelastic Relaxation

In this section, we assess whether viscoelastic relaxation of the crust could significantly affect the inferred stress evolution presented in the main text. In the energy-balance formulation, viscoelastic relaxation can be represented by a reduction in the fraction of work done by the pressurising source that is stored as elastic strain energy in the crust. In the main analysis this fraction is denoted by the parameter α (Methods; Eq. 10) and is taken as $\alpha = 1$, corresponding to the purely elastic end-member. Here we explore the implications of relaxing this assumption.

The importance of viscoelastic relaxation can be quantified using the Deborah number [4],

$$De = \frac{\tau_M}{\tau_{\text{obs}}}, \tag{S9}$$

where τ_{obs} is the characteristic timescale of the observed uplift episode and τ_M is the Maxwell relaxation time [5],

$$\tau_M = \frac{\eta}{G}, \tag{S10}$$

with η the effective viscosity and G the shear modulus. When $De \gg 1$, the relaxation timescale is long compared to the observation period and the crust behaves effectively elastically. Conversely, when $De \ll 1$, relaxation is expected to contribute significantly and time-dependent stress decay should be important.

Fig. S7.a shows De as a function of viscosity for the source geometries inferred at Campi Flegrei and Sierra Negra. Only for very low viscosities, corresponding to hot and weak crustal conditions [6], does De approach or fall below unity. For higher viscosities, representative of colder and mechanically stronger crust, $De \gg 1$, indicating that an elastic approximation is appropriate over the timescales considered here.

Rather than implementing a full viscoelastic forward model, we emulate the first-order effect of Maxwell relaxation on the elastic energy budget by allowing the effective fraction of source work stored as elastic strain energy, α (Methods; Eq. 10), to decay with time. In a Maxwell viscoelastic material, the constitutive relation implies that stress relaxes exponentially with characteristic timescale $\tau_M = \eta/G$; because elastic strain energy scales with stress (and strain), the stored elastic strain energy likewise decays exponentially [7]. We represent this behaviour by taking α to be time-dependent, such that

$$\alpha(t) = \exp\left(-\frac{t}{\tau_M}\right), \tag{S11}$$

which describes progressive dissipation of source work through time-dependent viscous creep. Applying this formulation to an imposed (approximately linear) uplift history causes the inferred elastic energy, and hence inferred pressure and stress, to deviate from the purely elastic prediction and tend towards a plateau at long times (Fig. S7.b).

To assess the potential influence of viscoelastic relaxation on our model results, we apply the exponential taper $\alpha(t)$ to the input energy histories for both Campi Flegrei and Sierra Negra (Fig. S7.c–d). We vary the effective viscosity over the range $\eta = 10^{19}$–10^{21} Pa s, which is representative of the mean viscosity of the shallow crust above a high-temperature pressure source [8] and for geothermal gradients comparable to those at both volcanoes [9, 10]. This approach implicitly treats the deforming crust as having a single bulk viscosity; within this framework, $\eta = 10^{19}$ Pa s represents a low (i.e., weak and hot) end-member value for the crust as a whole. In reality, even if localised regions of the crust are warm and deform viscously, the effective bulk viscosity controlling system-scale relaxation is expected to be higher. We find that, for both Sierra Negra and Campi Flegrei, the stress evolution including viscous relaxation converges toward the purely elastic model when $\eta > 10^{20}$ Pa s.

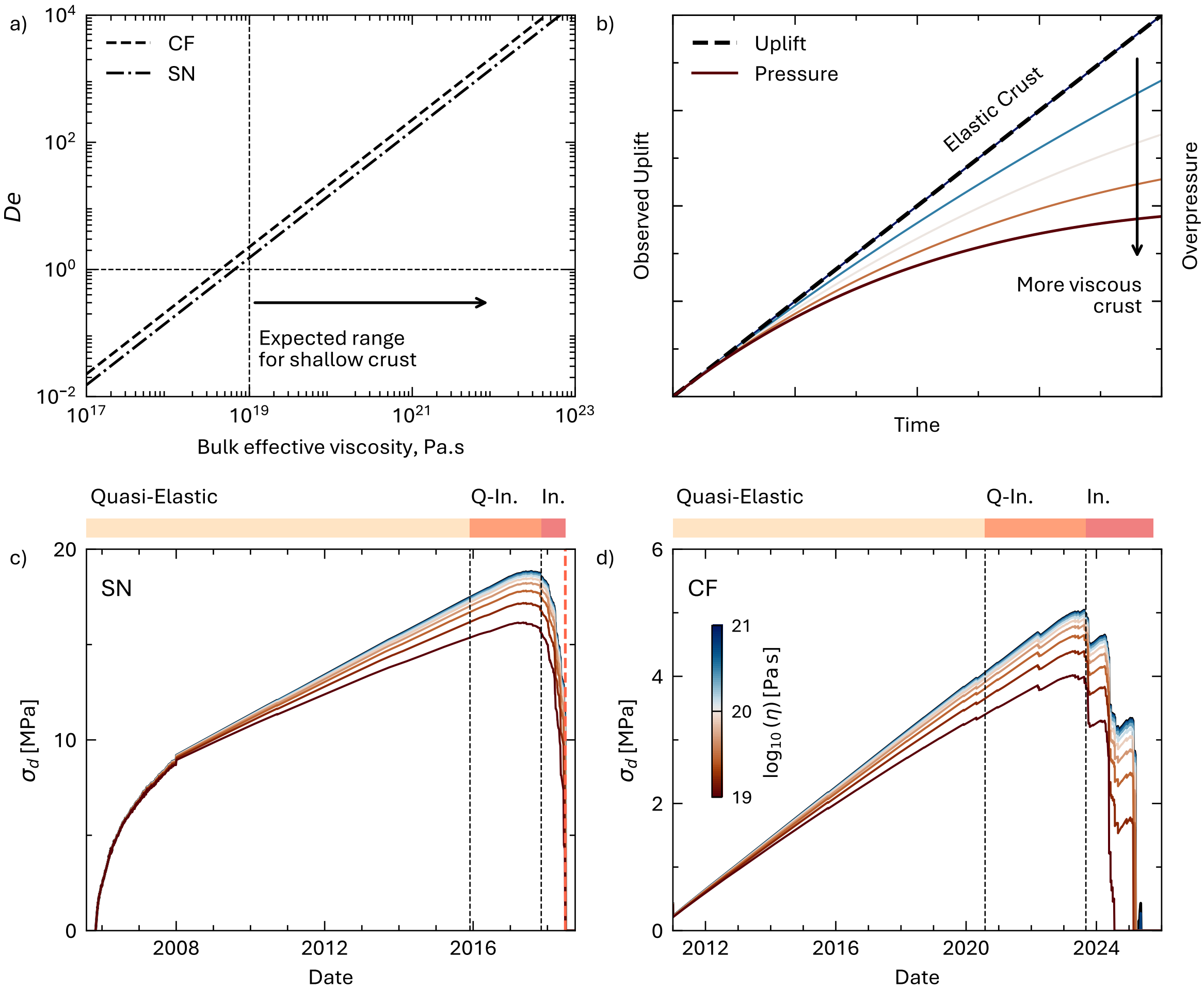

Figure S7: (a) Deborah number, $De = \tau_M/\tau_{\text{obs}}$, as a function of effective viscosity, η, for Campi Flegrei ($\tau_{\text{obs}} = 20$ yr; $G = 7$ GPa) and Sierra Negra ($\tau_{\text{obs}} = 10$ yr; $G = 16$ GPa), where $\tau_M = \eta/G$ is the Maxwell time. (b) Schematic illustration showing that viscoelastic relaxation causes the overpressure inferred from a linear uplift history to asymptote to a plateau relative to the purely elastic case. (c,d) Mean differential stress, σ_d, for Sierra Negra and Campi Flegrei recalculated for $\eta = 10^{19}$–10^{21} Pa,s using $\exp(-t/\tau_M)$ relaxation of elastic strain energy.

Increasing relaxation (i.e., reducing α) decreases the amplitude of the inferred stress, consistent with partial dissipation of elastic strain energy. Importantly, however, the overall temporal pattern of stress evolution remains unchanged: the transition from the initial quasi-elastic phase to the subsequent phase of enhanced energy release occurs at the same times across the explored parameter range, and the principal transition dates identified in the main analysis are unaffected. We therefore conclude that, even when viscoelastic relaxation is emulated in this manner, it does not materially change the key trends or timing of our results. Moreover, the widespread occurrence of volcano-tectonic seismicity during these episodes indicates that the crust does not behave as a purely viscous medium, and that elastic–brittle deformation remains an important component of the overall mechanical response. For these reasons, viscoelastic relaxation is omitted from the main-text figures and the main analysis retains the end-member assumption $\alpha = 1$.

References

[1] Fialko, Y., Khazan, Y. & Simons, M. Deformation due to a pressurized horizontal circular crack in an elastic half-space, with applications to volcano geodesy. *Geophysics Journal International* **146**, 181–190 (2001).

[2] Battaglia, M., Cervelli, P. F. & Murray, J. R. in *Modeling Crustal Deformation near Active Faults and Volcanic Centers - A Catalog of Deformation Models* 1 edn, Vol. 13 Ch. 1, 1–98 (USGS, 2013).

[3] McGarr, A. Some Comparisons between Mining-induced and Laboratory Earthquakes. *Pure and Applied Geophysics* **142**, 467–489 (1994).

[4] Degruyter, W. & Huber, C. A model for eruption frequency of upper crustal silicic magma chambers. *Earth and Planetary Science Letters* **403**, 117–130 (2014).

[5] Dragoni, M. & Magnanensi, C. Displacement and stress produced by a pressurized, spherical magma chamber, surrounded by a viscoelastic shell. *Physics of the Earth and Planetary Interiors* **56**, 316–328 (1989).

[6] Zhan, Y. & Gregg, P. M. How Accurately Can We Model Magma Reservoir Failure With Uncertainties in Host Rock Rheology? *Journal of Geophysical Research: Solid Earth* **124**, 8030–8042 (2019).

[7] Head, M., Hickey, J., Gottsmann, J. & Fournier, N. The Influence of Viscoelastic Crustal Rheologies on Volcanic Ground Deformation: Insights From Models of Pressure and Volume Change. *Journal of Geophysical Research: Solid Earth* **124**, 8127–8146 (2019).

[8] Head, M., Hickey, J., Gottsmann, J. & Fournier, N. Exploring the Impact of Thermally Controlled Crustal Viscosity on Volcanic Ground Deformation. *Journal of Geophysical Research: Solid Earth* **126**, e2020JB020724 (2021).

[9] Gregg, P. M. *et al.* Forecasting mechanical failure and the 26 June 2018 eruption of Sierra Negra Volcano, Galápagos, Ecuador. *Science Advances* **8**, eabm4261 (2022).

[10] Castaldo, R. *et al.* The impact of crustal rheology on natural seismicity: Campi Flegrei caldera case study. *Geoscience Frontiers* **10**, 453–466 (2019).
