# Supplementary material for: Crustal weakening before rupture at volcanoes after long repose
Source: Nat Commun. 2026 Aug 24;17:10152. doi: 10.1038/s41467-026-77001-5 (PMC13601623; doi:10.1038/s41467-026-77001-5)
Supplement: Supplementary file 2 [file 41467_2026_77001_MOESM2_ESM.pdf]

nature portfolio

Peer Review File

Crustal weakening before rupture at volcanoes after long repose

Corresponding Author: Dr Eric Newland

This file contains all reviewer reports in order by version, followed by all author rebuttals in order by version.

Version 0:

Reviewer comments:

Reviewer #1

(Remarks to the Author)
This manuscript is thought-provoking and took many days to organize this review report.

This study introduces a new method of delineating temporal changes in stored energy in the crust during volcanic unrest. The manuscript is well-organized and self-consistent. The logic is easy to follow. However, I have some concerns about this study; some are major and minor, as enumerated below. I want the authors to address these comments before this study is considered for publication.

1. The main conclusion of this study is that the acceleration of deformation is due to the weakening of the crust by a series of earthquakes rather than an accelerated magma supply. While the hypothesis that the magma supply is constant over time makes sense, observations do not support the authors' conclusion. The authors infer that the damage during unrest in Sierra Negra and Campi Flegrei decreased the rigidity by 18 and 30 percent, respectively (Line 177). Because the rigidity is proportional to the square of the S-wave velocity, assuming the constant density, 18 and 30 percent reductions of rigidity correspond to about 9.5 and 16.3 percent of the bulk S-wave velocities, respectively. However, recent studies of seismic interferometry, for example, have detected velocity changes of 1 to a few percent at most. An exception is Nakata & Snieder (2011, GRL), who detected ~10 percent of S-wave velocity reduction by the strong shaking of the 2011 Tohoku-oki earthquake. The velocity reduction is localized at the unconsolidated sediment at the shallowest 100 meters. Therefore, volcanic earthquakes cannot reduce the bulk rigidity of Sierra Negra and Campi Flegrei by >18 percent. Although a significant rigidity reduction is possible only around the area of seismic activity, a bulk rigidity reduction of >18 percent is required to meet the authors' hypothesis.

2. Line 53: The Results section describes seismic and geodetic observations in Sierra Negra and Campi Flegrei. This part should be an independent section of "Observation" or something similar.

3. The labels of the panels in Figure 1 are confusing. I recommend the authors to label the panels as a, b, c, d, e, f, rather than a.i, a.ii, a.iii, b.i, b.ii, b.iii.

4. Similarly, panels in Figures 4, 5, 6, and 7 should be revised.

5. Line 68: The location of GS09 and VCH1 should be shown in a figure.

Reviewer #2

(Remarks to the Author)
Although it is a novel idea, this study is fundamentally flawed, both conceptually and in how the calculations are managed. I do not see an avenue to publication, especially in Nature. Although we cannot model the crust perfectly and always must make assumptions, the assumptions in this study are simply too numerous and overly simplistic to justify.

1. The role of gas is completely neglected in this study. Gas overpressure drives volcanic eruptions. As earthquakes occur above an intrusion, they affect not only the stress field. They also increase permeability allowing magma to degas. Thus, increased fracturing can decrease the chance of eruption by allowing degassing. This study assumes that the role of gas flux

is negligible, which is inconsistent with most of what we know about evolution of volcanic systems. There are eruptions in which gas is contained to the point that the gas release story is secondary compared to accelerating seismicity, but that is rare.

2. The calculations do not make sense to me. For Ue the authors calculate stored strain energy (stress change) in the crust due to radiated seismic waves early in the sequence without accounting for the fact that those same earthquakes are relieving stress as well. Both things occur with every earthquake. In addition, by using radiated seismic energy as a proxy for stress change the authors neglect other known ways in which earthquakes stress the surrounding crust. It is well established that earthquakes cause both static and dynamic stress changes that can be estimated (to an order of magnitude only) with detailed information on the earthquake's location and radiation pattern. Radiated seismic energy (used here) would be a better estimate for dynamic stress changes, but the authors seem to be interpreting it as a static stress change. If they believe it is a good proxy for static stress change, how does it compare to Coulomb stress change calculations?? This should be shown to justify the approach.

3. Distance from the earthquake is critical when calculating stress change due to earthquakes (either imparted by seismic waves or relieved). This is true even over short distances for small earthquakes, and if large earthquakes occur nearby but outside the radius defined in this study (eg. a M 6+ within 100 km or so), those earthquakes may change the stress in the crust above the magmatic intrusion more than the smaller local earthquakes will. All of this is neglected in this study.

4. The study does not consider crustal relaxation, which is known to occur in the stable shallow crust over the time scales considered in this study. There is a rich literature on this subject. If the time scales considered were short (weeks), the authors could potentially avoid accounting for relaxation, particularly for small earthquakes.

5. Although we are forced to use the homogenous elastic medium model in many cases to model seismic and deformation data, it is almost certainly not true around magma chambers and above a large caldera, in particular. The authors note this and refer to it as 'quasi-elastic' yet proceed with the assumption in modeling the deformation data and in other aspects of the study. There is extensive literature showing that seismicity is often much shallower than magma is inferred to be in volcanic systems due to there being an aseismic ductile layer under an impermeable boundary, including at Campi Flegrei (see ML high precision relocation/catalog enhancement work led by Xing Tan of Stanford. This should be published withing weeks, but is already available in abstracts). The role of that ductile layer in modifying the stress field due to intrusion should not be neglected. The cartoon in figure 7 itself is inconsistent with a homogeneous elastic model since it assumes the earthquakes are occurring on weak surfaces – the caldera ring faults. It is reasonable to assume an elastic medium in many cases, but not to calculate a time series of stress changes that has multiple orders of magnitude of error then interpret it in detail.

6. The concept that the crust switches from earthquakes being related to increasing stress then to decreasing strength after crossing a specific threshold hinges on the value of Uo in equation 5 in relation to the seismic energy. Equation 5 is a product of P and dV, yet in a magma body these two terms are dependent on each other. The calculations for P and dV are oversimplified to an unreasonable degree (or not well explained), as the role of gas and fluid pressure is neglected in this study.

7. I find it confusing that the authors use an elastic model for all the initial calculations, then use the results of those calculations to infer a shift in the crust from elastic to inelastic. This seems circular.

8. Errors are not discussed. It is well known in the seismic source process literature that seismic efficiency, stress drop, estimates of radiated energy etc can vary by orders of magnitude between individual earthquakes, yet the authors cite none of this literature. Similarly, there are inevitably large errors in the geodetic modeling done here. Error sources need to be very seriously considered in this and all research.

9. Line 349: "Since volcanoes primarily deform by changing their shape [2], we assume that the strain energy calculated in Eq. (8) represents the distortional component of strain energy". This doesn't make sense to me. Volumetric changes occur within and above magmatic systems regularly and pore pressures changes are a critical part of the stress regime near volcanoes.

10. The examples given are not adequate to support the method. It would be better to apply this at volcanoes that have multiple eruptions and long-time histories of quality seismic and deformation data, as the false positive rate must be assessed to fully support the method, particularly as b-values are heavily weighted in assessing the model in the paper. The authors are using the same data set (earthquake catalog) to both calculate stress changes and assess how good the calculations are. Again, there is a potential for circular reasoning.

11. Throughout the manuscript, except for one section of the methods, "stress" is discussed as if it is a scalar. Stress is a tensor and the type of stress and the surfaces on which that stress acts needs to be defined specifically throughout the manuscript. The potential interaction between inferred stresses and a regional stress field also deserves a mention. Finally, "Stress" and "strain" are used in a way that reads in portions of the paper as if they're interchangeable, and they are not.

12. Several statements in the introduction simplify the state of eruption forecasting based on seismicity to the point of being incorrect. Because most eruptions occur at open system volcanoes (the frequently erupting volcanoes), most eruptions do not have earthquakes that accelerate into eruption. Also it has long been known that a transition from VT earthquakes to tremor and long period earthquakes is critical for eruption forecasting – a much more nuanced analysis than what can be done with earthquake catalogs alone. Earthquakes accelerate before some eruptions, but it is certainly not all and not the

majority.

13. The point above is particularly true for caldera systems which are known to have complex precursory sequences with multiple swarms before erupting (see work by Acocella and Newhall). Also caldera systems (including Campi Flegrei) after have very active hydrothermal systems. These have their own seismicity which reflects shallow hydrothermal processes rather than deep intrusion.

Reviewer #3

(Remarks to the Author)
This paper presents an innovative approach to monitoring volcanic unrest by analyzing changes in crustal stress and elastic strain energy. The calculated energy evolution is then compared to the energy released by VT earthquakes. The study focuses on two caldera systems, Sierra Negra (Galápagos) and Campi Flegrei (Italy), to demonstrate how variations in stored elastic strain energy and seismicity patterns can predict transitions from elastic crustal deformation to inelastic, fracture-driven weakening, ultimately leading to eruptions. Notably, the research connects decreasing stored stress to crustal weakening during volcanic unrest, challenging the conventional assumption that increasing stress directly correlates with seismicity. An increase in VT event rates under decreasing stress is thus identified as a crucial new criterion for recognizing the approach of crustal rupture and a possible eruption.

The paper is well-written, and I enjoyed reading it. The presentation of the results is of high quality. Although the calculation of stress and energy may not achieve sufficient accuracy for precise forecasting of volcanic eruptions, I believe the manuscript is worth publishing and will generate significant interest within the research community.

The major issue with the paper lies in its determination of the different deformation stages (i.e., elastic, inelastic), which relies heavily on the assumption of when the linear stage of uplift begins and ends. Different assumptions could lead to significantly different results. For example, the long-term deformation of Campi Flegrei does not clearly exhibit a stage of uplift with a constant rate. Instead, one could describe the deformation history more accurately using a power law. Therefore, addressing the non-uniqueness of this interpretation is crucial.

Moreover, the authors have not fully accounted for crustal relaxation, the complexities of the magma system, or variations in magma overpressure. Some scientists may argue that the overpressure may not evolve linearly, even with constant magma injection, as assumed in the paper. That said, I do agree with the authors that the final stage of crustal damage—marked by accelerating surface deformation accompanied by increasing seismicity—could signal the formation of a major through-going fracture, which may lead to an eruption. This is a strong and valuable conclusion. Therefore, I recommend that the authors carefully address the uncertainties surrounding different interpretations of deformation patterns. They should evaluate whether the proposed framework remains robust and applicable under alternative scenarios. This would significantly strengthen the validity and utility of their methodology.

Finally, the deformation volume plays a critical role in determining the magnitude of the estimated energy. I conducted a simple numerical simulation of a magma chamber (long axis = 3000 m, short axis = 600 m, depth = 3000 m, and pressure change = 30 MPa). The resulting total elastic strain energy change in the crust was on the order of 10^15 J, which appears consistent with the authors' estimations. However, it is unclear whether the simplified estimation used in Equation (1) is valid for different magma chamber geometries. Additionally, the choice of deformation volume is problematic. In real cases, deformation theoretically occurs over an infinite volume, making it challenging to define a finite deformation region. It would be beneficial for the authors to address this issue explicitly or, alternatively, use numerical modeling to benchmark their calculations. This approach would help validate the robustness of their framework across varying chamber geometries and deformation scenarios.

Some minor comments:

In the introduction, it is worth noting that many studies have already explored the relationship between elastic energy during deformation and seismicity. For example, Figure 9 and the discussion in https://doi.org/10.1029/2020JB020158 provide valuable insights into this topic. However, these studies have struggled to reconcile the magnitude of energy released by fracturing and seismicity. The primary issue is that fracturing releases only a small fraction of the stored elastic energy, whereas earthquakes release a significantly larger portion of this energy. This suggests that fracturing and damage within the crust may serve as a trigger rather than the primary mechanism for energy release. In this context, while the authors' framework offers a novel perspective, it would benefit from further discussion of this disparity between fracturing and seismic energy release. Addressing this point would strengthen the argument and provide a more comprehensive understanding of the processes leading to volcanic eruptions.

Line 35: "Increases in VT event rate may thus be driven initially by an increase in stress and later by a decrease in the crust's strength." This statement implies that a drop in crustal strength leads to the generation of earthquakes, which is conceptually incorrect. Instead, the drop in crustal strength is a consequence of earthquakes and fracturing processes, rather than a driving force for seismicity. It would be more accurate to clarify that the weakening of the crust, caused by fracturing and prior seismic activity, facilitates further fault slip and deformation, which may indirectly accelerate seismicity. This distinction is important for accurately describing the relationship between crustal strength and earthquake generation.

Version 1:

Reviewer comments:

Reviewer #1

(Remarks to the Author)
The authors have done a very good job in addressing the comments from the original version. I am now satisfied with the manuscript, and I am happy to recommend the acceptance of this study as is.

Reviewer #3

(Remarks to the Author)
The authors propose an energy-balance framework that tracks the elastic strain energy stored in volcanic crust as the difference between energy supplied by a pressurising source and energy lost through VT seismicity. The key result is that seismicity and uplift can accelerate even while mean differential stress decreases, because fracture-induced weakening lowers the crust's resistance — a signal of approaching rupture. The method is tested against Sierra Negra (a complete pre-eruptive sequence) and Campi Flegrei (ongoing).

I thank the authors for their careful and thorough response. I am pleased to confirm that the concerns I raised in my previous review have been fully addressed. In particular, the non-uniqueness of the deformation-stage determination — my principal concern — is now explicitly acknowledged in the main text and tested in a new sensitivity analysis (Appendix B) using alternative power-law uplift parameterisations, which confirms that the inferred regime transitions are robust. The role of crustal relaxation is now treated quantitatively via the Deborah number and a Maxwell-decay analysis (Appendix D), and the conceptual point regarding crustal strength as a consequence rather than a driver of seismicity has been corrected. The additional geodetic-versus-seismic moment comparison further strengthens the physical justification of the approach. These revisions have materially improved the manuscript.

More broadly, this is a clear, well-written, and genuinely novel contribution. The idea that accelerating seismicity need not imply increasing stress has direct relevance to interpreting monitoring data during crises, and the three-regime structure (quasi-elastic → quasi-inelastic → inelastic) is intuitive and useful. That the same evolution emerges at two volcanoes with very different crustal and hydrothermal settings is convincing evidence of a first-order process.

The work is methodologically careful and appropriately cautious: the authors stress that the method tracks relative temporal changes, not absolute stress, and they back this up with strong supporting analyses. Appendix A benchmarks the simplified energy estimate against a full Fialko-model calculation and defines a finite deformation volume; Appendix C propagates uncertainty in shear modulus and seismic efficiency via Monte Carlo. In each case the timing of transitions is robust, and b-values are correctly used as a posteriori corroboration rather than as model input.

In summary, my earlier concerns have been satisfactorily resolved. The manuscript represents a clear conceptual advance, supported by a transparent method and rigorous uncertainty analysis, with claims kept appropriately modest. The remaining issues are cosmetic. I recommend acceptance after minor corrections.

Minor Points
1. Appendix B figure is labelled "Figure 11" but referred to as "Fig. D1a/b" in the text — reconcile.
2. Viscosity range is inconsistent: text says 10^19 – 10^21 Pa*s, Fig. 14 caption says 10^18 – 10^21
3. A few typos remain (e.g., there are two "robust" in Line-280 and "propagarte" in Line-599).

Reviewer #4

(Remarks to the Author)
Review: Crustal Weakening as a Precursor to Volcanic Eruption after Long Repose
This paper discusses how the crust above a pressure source behaves during accelerating unrest. I think that the topic is important and I think the authors have come up with a reasonable model to bring together seismic and geodetic observations. The structure of the paper and the writing is good. The paper was (thankfully) reviewed by individuals that know much more about modeling crustal processes than I do. My expertise is in the seismicity, which is what most of my comments relate to. Individual comments are below, but here are a couple of broader points:

1) I wish that the authors had chosen another volcano that actually erupted. The framing of this as a precursory tool and then including Campi Flegri (CF) leads one to believe that CF is about to erupt. The authors may believe that. If they keep at it, eventually they will be correct. It is unreasonable to ask to analyze another volcano (Mauna Loa, Okmok, Piton de la Fournaise all come to mind) so I highly suggest softening the language throughout the paper to keep the results here from looking like a forecast.

2) I think the authors put too much emphasis on the b-value. Admittedly, I don't like b-values at all because they are too susceptible to subtle changes in the earthquake catalog and the windows over which they are calculated. B-values are not without value, when used with other parameters. At times, the authors describe the b-values as they are presented conceptually in Figure 8, and not how they actually look with real data in Figure 7. B-values are messy.

In the end, the paper is definitely worthy of publication after some minor adjustments to the writing.

Line 17-19: A little too colloquial for my taste, but it's up to you.
Line 21: What other signals? Cast somewhat differently, changes in VT rates are common during unrest, but it is hard to know in the moment what the VT rate (or energy release) is that will lead to an eruption.
Line 24: Grouping reference 8 in with deterministic modeling is a stretch

Line 74: "rapid increase of 1.77 m until June 2007". Is there an error in units here, or maybe "uplift" should be used instead of "increase"? 1.77m from what?

Line 79-81: I assume to get a location you need more than these two stations, so I'm not sure why they are being called out here. Or maybe these are actually event counts (the dN/dt in Figure 1)?
Line 82: Bell uses Mc=1.2 by my reading of the paper.
Line 83-85: The rates appear to be much lower than 830 events/month and seem to be accelerating through 2017 and 2018.

Line 152: Apologies if I'm being dense here, but I've read the methods and the main text several times and can't figure out how the b-value is incorporated into the model.

Line 201: If there were station outages at SN, how do you know that the VT seismicity was low during the initial adjustment phase? Is it worth noting that the maximum magnitude is trending generally higher during this time?

Line 208: Based on Figure 1, I don't see a significant difference in the seismicity, as stated in the text, until well after the end of the quasi-elastic phase. Is it worth an inset to show the difference?

Line 213-214: Figure? This analysis depends greatly on your window size. I don't dispute that there is a change in seismicity…eventually…only that it isn't obvious from figure 1 to be at the vertical line.

Line 216-218: Would love to see a figure of this. At the scale of Figure 1 (and Figure 7), it isn't obvious.

Line 219-222: I'm not sure what this paragraph is referring to. Consider a transition sentence in the paragraph before, or a better topic sentence.

Line 280: "robust" is repeated

Line 284-285: I definitely see the magnitudes increase through the quasi-elastic phase…especially at CF. This sentence seems to say two things at once.

Line 290: No need to use "4.71", nobody knows the magnitude to that precision.

Line 330: "constant maximum magnitudes" is disputable

Line 330: "oscillations in b-value about a stable mean": for SN…maybe but for CF the mean doesn't have any utility because of how variable the b-value is. The use of oscillations and stable in the same sentence is also confusing.

Line 332-334: I like this interpretation a lot, but while the b-values were stable (in part because of the number of events) it doesn't seem like they are meaningfully less than some periods during the quasi-elastic phase.

Line 341-343: there are a ton of changes in the b-value during all phases except the inelastic phase and thus I don't think it is very good precursor, unless you are suggesting combining it with something else, in which case this sentence needs to be clarified.

Line 366: I'm uncomfortable with the use of precursory as it pertains to CF. Is it before the next eruption…yes. But until CF erupts, it is hard to tell whether this is actually precursory activity that portends an eruption or not.

Line 371: What about new magma inputs into the system?
Line 371: Have you considered healing of the crust that may occur during repose. Seems like it may have an effect: Thelen et. al, 2010 10.1029/2010GL044194.

Line 389: I would challenge the "real-time" applicability here. Precise GNSS locations are hardly real time, as are quality estimates of earthquake magnitude. Deciding when to stop assuming that a linear trend is being followed takes several abnormal GNSS locations. The analyses here require long time windows to generate b-values and rates over, which are slow to change. I don't necessarily dispute the premise of the sentence, only its applicability in real time or even near real time.

Line 393: This paper seems relevant: (Hill, 1977) 10.1029/JB082i008p01347.

Line 422: is there a reference for this statement?
Line 424: Can you briefly describe how you would take into account the Ur term in an area where tectonics do play a role?

Can the red line in Figure 5,6,7 be made thicker?

Figure 1: The units on dN/dt are awkward.

Figure 4: Consider moving to the methods section?

Figure 5: Colors could be more contrasted to be more easily interpretable. It's also confusing that you are using the same colors for different things in the same figure.

Figure 8: I really like this figure. In c) the way that the swarm-like seismicity is drawn, I would expect an increase in b-value during swarms (smaller fault planes creating lower magnitude earthquakes), which is not obvious in the analyses.

Open Access This Peer Review File is licensed under a Creative Commons Attribution 4.0 International License, which permits use, sharing, adaptation, distribution and reproduction in any medium or format, as long as you give appropriate credit to the original author(s) and the source, provide a link to the Creative Commons license, and indicate if changes were made.
In cases where reviewers are anonymous, credit should be given to 'Anonymous Referee' and the source.
The images or other third party material in this Peer Review File are included in the article's Creative Commons license, unless indicated otherwise in a credit line to the material. If material is not included in the article's Creative Commons license and your intended use is not permitted by statutory regulation or exceeds the permitted use, you will need to obtain permission directly from the copyright holder.
To view a copy of this license, visit https://creativecommons.org/licenses/by/4.0/

Referee response: Crustal Weakening as a Precursor to Volcanic Eruptions after Long Repose

Eric Newland & Christopher Kilburn

We thank the reviewers for their constructive comments on our manuscript. We are grateful for the time they spent to evaluating the work. Their suggestions have helped us to improve the clarity, robustness, and scope of the paper. Below, we provide a detailed point-by-point response and outline the revisions made in the manuscript.

Reviewer 1:

1. The main conclusion of this study is that the acceleration of deformation is due to the weakening of the crust by a series of earthquakes rather than an accelerated magma supply. While the hypothesis that the magma supply is constant over time makes sense, observations do not support the authors' conclusion. The authors infer that the damage during unrest in Sierra Negra and Campi Flegrei decreased the rigidity by 18 and 30 percent, respectively (Line 177). Because the rigidity is proportional to the square of the S-wave velocity, assuming the constant density, 18 and 30 percent reductions of rigidity correspond to about 9.5 and 16.3 percent of the bulk S-wave velocities, respectively.

However, recent studies of seismic interferometry, for example, have detected velocity changes of 1 to a few percent at most. An exception is Nakata & Snieder (2011, GRL), who detected 10 percent of S-wave velocity reduction by the strong shaking of the 2011 Tohoku-oki earthquake. The velocity reduction is localized at the unconsolidated sediment at the shallowest 100 meters. Therefore, volcanic earthquakes cannot reduce the bulk rigidity of Sierra Negra and Campi Flegrei by >18 percent. Although a significant rigidity reduction is possible only around the area of seismic activity, a bulk rigidity reduction of >18 percent is required to meet the authors' hypothesis.

- Thank you for this comment. In our framework, the modulus represents an effective bulk property of the deforming volume, integrating the mechanical influence of pre-existing discontinuities and damage accumulation during unrest, rather than a direct prediction of spatially uniform seismic velocity change. We have therefore removed the section quantifying the value of D, as this parameter is model-dependent and not central to our main argument. Our conclusion does not require a literal bulk reduction in rigidity of the magnitude discussed above, but rather that progressive damage reduces the effective mechanical resistance of the deforming crust sufficiently to modify the deformation response under continued pressurisation. We now clarify this distinction in the revised manuscript.

2. Line 53: The Results section describes seismic and geodetic observations in Sierra Negra and Campi Flegrei. This part should be an independent section of "Observation" or something similar.

- We agree that separating observations from interpretation can improve clarity. However, the journal's prescribed manuscript structure does not allow an "Observations" section as a stand-alone heading.

3. The labels of the panels in Figure 1 are confusing. I recommend the authors to label the panels as a, b, c, d, e, f, rather than a.i, a.ii, a.iii, b.i, b.ii, b.iii.
4. Similarly, panels in Figures 4, 5, 6, and 7 should be revised.
5. Line 68: The location of GS09 and VCH1 should be shown in a figure.

- Done

Reviewer 2:

1. The role of gas is completely neglected in this study. Gas overpressure drives volcanic eruptions. As earthquakes occur above an intrusion, they affect not only the stress field. They also increase permeability allowing magma to degas. Thus, increased fracturing can decrease the chance of eruption by allowing degassing. This study assumes that the role of gas flux is negligible, which is inconsistent with most of what we know about evolution of volcanic systems. There are eruptions in which gas is contained to the point that the gas release story is secondary compared to accelerating seismicity, but that is rare.

- We agree that gas exsolution, transport, and flux can play a key role in volcanic unrest and eruption, and that fracturing may increase permeability and promote degassing. Our study does not assume that gas transport is absent or unimportant. We do not attempt to model volatile transport, permeability changes or degassing explicitly; instead, we interpret the observed surface deformation in terms of pressurisation within a shallow reservoir. We make no assumption about the nature of the pressurising fluid, which could be either gas or magma, and we do not seek to constrain the mechanism driving pressurisation. Indeed, our interpretation is compatible with concurrent degassing, provided that the net balance of pressurised fluid in the source remains positive over the analysed interval (i.e., supply to the storage region exceeds release). We have clarified this in the Methods.

2. The calculations do not make sense to me. For Ue the authors calculate stored strain energy (stress change) in the crust due to radiated seismic waves early in the sequence without accounting for the fact that those same earthquakes are relieving stress as well. Both things occur with every earthquake. In addition, by using radiated seismic energy as a proxy for stress change the authors neglect other known ways in which earthquakes stress the surrounding crust. It is well established that earthquakes cause both static and dynamic stress changes that can be estimated (to an order of magnitude only) with detailed information on the earthquake's location and radiation pattern. Radiated seismic energy (used here) would be a better estimate for dynamic stress changes, but the authors seem to be interpreting it as a static stress change. If they believe it is a good proxy for static stress change, how does it compare to Coulomb stress change calculations?? This should be shown to justify the approach.

- Our objective is not to estimate the static stress change (or stress drop) on individual fault planes, nor to infer a Coulomb stress perturbation in the surrounding crust. Rather, we use earthquake energetics to estimate the amount of elastic strain energy released by each event, and then track the cumulative energy release through time.

  We start from the radiated seismic energy, E_r, (as is it a commonly estimated parameter) and convert it to an estimate of the total elastic strain energy change associated with the earthquake using the seismic efficiency [1–3], which accounts for the fact that only a fraction of the released elastic strain energy is radiated as seismic waves (with the remainder dissipated by inelastic processes such as frictional heating, damage, and other losses). In this framework, earthquakes are treated as an energy sink that reduce stored elastic strain energy; we are not adding strain energy to the crust via seismic waves (which would account for dynamic stress changes).

  Because our metric is based on a bulk energy balance (and, in places, an energy-derived equivalent crustal stress measure obtained by distributing energy over a representative crustal volume), it is not directly comparable to Coulomb stress change calculations, which quantify spatially heterogeneous static stress perturbations that depend on detailed fault geometry and slip distribution. Radiated energy also primarily reflects the dynamic wavefield, but we do not interpret it as a proxy for a local static stress change; instead, it is used as an observable that can be related to the earthquake's total energy budget through seismic efficiency.

We have therefore expanded and clarified the methodological description in the revised manuscript to make these distinctions explicit [lines 460-470].

3. Distance from the earthquake is critical when calculating stress change due to earthquakes (either imparted by seismic waves or relieved). This is true even over short distances for small earthquakes, and if large earthquakes occur nearby but outside the radius defined in this study (eg. a M 6+ within 100 km or so), those earthquakes may change the stress in the crust above the magmatic intrusion more than the smaller local earthquakes will. All of this is neglected in this study.

- Large-magnitude earthquakes may produce both dynamic and static stress perturbations within our control volume. Our framework is not intended to compute spatially resolved stress changes from individual earthquakes. Instead, we track a bulk energy budget in which VT earthquakes represent an energy sink (released elastic strain energy) and source pressurisation represents an energy input. In this context, dynamic shaking would most plausibly appear as a transient perturbation superimposed on the background energy state, whereas a static stress perturbation could produce an impulsive offset (step-like change) in the inferred background stress and energy states.

  These effects are expected to be short-term disturbances rather than a sustained change in the pressurisation-driven evolution that controls the multi-year trends analysed here. Because our interpretation relies primarily on relative changes in the rates of energy supply and cumulative energy loss through time, such perturbations are unlikely to bias the key temporal patterns unless they are large and/or repeated. We therefore treat external earthquake effects as a secondary contribution in this first-order formulation, while noting that explicit modelling of large nearby events would be a useful extension where such earthquakes occur during the analysed interval.

  We have added a short description of these effects in the Discussion [lines 373-390].

4. The study does not consider crustal relaxation, which is known to occur in the stable shallow crust over the time scales considered in this study. There is a rich literature on this subject. If the time scales considered were short (weeks), the authors could potentially avoid accounting for relaxation, particularly for small earthquakes.

- We have added a new Appendix D on viscoelastic relaxation in which we (i) quantify the expected importance of relaxation using the Deborah number, $De = \tau_M/\tau_{obs}$, with $\tau_M = \eta/G$ representing the Maxwell time [4], and (ii) test the first-order impact of Maxwell relaxation on our inferred energy/stress histories. We represent relaxation by applying an exponential decay, $\exp(-t/\tau_M)$ to the elastic strain-energy input [5] and recompute the stress evolution for a wide range of effective viscosities ($\eta = 10^{19}$–10^{21} Pa s). TThese viscosities represent plausible mean effective values for the shallow crust above a high-temperature reservoir [6], with geothermal gradients comparable to those at both volcanoes [7, 8].. This sensitivity analysis shows that increasing relaxation reduces the amplitude of inferred stresses, as expected, but does not change the key temporal patterns or the timing of the main transition(s) identified in the paper. We therefore conclude that neglecting relaxation in the main-text figures does not materially affect our principal conclusions, while noting that explicit viscoelastic modelling would be a useful extension where independent constraints indicate very low effective viscosities, which are unrealistic in the current examples.

5. Although we are forced to use the homogenous elastic medium model in many cases to model seismic and deformation data, it is almost certainly not true around magma chambers and above a large caldera, in particular. The authors note this and refer to it as 'quasi-elastic' yet proceed with the assumption in modelling the deformation data and in other aspects of the study. There is extensive literature showing

that seismicity is often much shallower than magma is inferred to be in volcanic systems due to there being an aseismic ductile layer under an impermeable boundary, including at Campi Flegrei (see ML high precision relocation/catalog enhancement work led by Xing Tan of Stanford. This should be published withing weeks, but is already available in abstracts). The role of that ductile layer in modifying the stress field due to intrusion should not be neglected. The cartoon in figure 7 itself is inconsistent with a homogeneous elastic model since it assumes the earthquakes are occurring on weak surfaces – the caldera ring faults. It is reasonable to assume an elastic medium in many cases, but not to calculate a time series of stress changes that has multiple orders of magnitude of error then interpret it in detail.

- We agree that the crust beneath large volcanic edifices and calderas is mechanically heterogeneous and may include thermally weakened and ductile regions, so a homogeneous elastic medium is an approximation. Our use of the term "quasi-elastic" is intended to acknowledge this: we do not assume the crust is perfectly elastic everywhere, but rather that, during the early stage of unrest, the observed surface deformation can be approximated by an effective elastic response.

  To address the concern that heterogeneity and time-dependent rheology could lead to large errors in inferred stress and energy, we have added two new analyses. First, as described in detail in our response to query 4, we have added a new Appendix D (Viscoelastic Relaxation) that evaluates the potential importance of Maxwell relaxation over the observation timescales using the Deborah number and a first-order relaxation emulation applied to the input energy histories. Second, Appendix B (Uncertainty in Energy and Stress Estimates) propagates key uncertainties through the full stress-evolution model using a Monte Carlo approach. In particular, we treat the shear modulus as a random variable (normally distributed about plausible shallow-crust values) to represent uncertainty in the effective bulk stiffness expected from layered and heterogeneous structures. We then recompute the full stress evolution across 1000 realisations and report ensemble means and uncertainty envelopes. These analyses show that uncertainty in the model parameters affects the absolute magnitudes of the inferred stresses, but does not alter the key temporal patterns or the timing of the main transition(s) identified in the paper.

  We also wish to clarify the point regarding Figure 7. We agree that VT earthquakes at calderas commonly localise on pre-existing weak structures (e.g., ring faults), and Figure 7 is intended as a conceptual illustration of this localisation rather than a literal depiction of a homogeneous medium at the scale of individual faults. Importantly, our energy-balance framework does not require earthquakes to be spatially distributed within a homogeneous elastic half-space, nor does it rely on calculating event-by-event stress changes at specific locations. The seismic catalogue enters the analysis only through the cumulative energy released by VT events (converted to released elastic strain energy using a seismic-efficiency distribution). Because this metric is volume-integrated and energy-based, the spatial distribution of earthquakes (whether diffuse or localised on weakened planes) does not affect the inferred energy-release time series, provided the catalogue captures the relevant events.

  Finally, we agree that absolute stress magnitudes inferred from simplified elastic representations should not be over-interpreted. The new sensitivity tests demonstrate that uncertainties and heterogeneity primarily affect the absolute scaling of inferred stress and energy, whereas the temporal evolution central to our interpretation are robust across plausible parameter ranges. We therefore further emphasise in the manuscript that the method is best used to track relative temporal evolution and transition timing, rather than absolute magnitudes. We have updated the manuscript accordingly and added Appendices B and C to address this comment.

6. The concept that the crust switches from earthquakes being related to increasing stress then to decreasing strength after crossing a specific threshold hinges on the value of Uo in equation 5 in relation

to the seismic energy. Equation 5 is a product of P and dV, yet in a magma body these two terms are dependent on each other. The calculations for P and dV are oversimplified to an unreasonable degree (or not well explained), as the role of gas and fluid pressure is neglected in this study.

- Our interpretation does not assume that P and ΔV are independent in a magma reservoir. In an elastic source model the two are linked through the source compliance (for a given geometry and elastic structure, $\Delta V \propto P$), and the expression $U_o = (1/2)P\Delta V$ is the standard work/strain-energy result for linear elastic pressurisation [9, 10]. Thus, Eq. (5) does not treat P and ΔV as separate free variables; they are two equivalent parameterisation of the same pressurisation process.

  More importantly, the transition we identify is not tied to a single threshold value of U_o, but to the time-evolution of the energy budget: the relative rates of energy input inferred from deformation and energy release inferred from VT seismicity. While uncertainties in source geometry and elastic stiffness affect the absolute scaling of P, ΔV, and U_o, our sensitivity tests (Appendix B) show that the temporal patterns and transition timing are robust across plausible parameter ranges. Finally, as stated in our response to query 1, we do not assume zero volatile flux; rather, we interpret the deformation signal as net pressurisation without explicitly modelling degassing/volatile transport, which we now clarify in the Methods.

7. I find it confusing that the authors use an elastic model for all the initial calculations, then use the results of those calculations to infer a shift in the crust from elastic to inelastic. This seems circular.

- We agree the description can be clarified, but the inference is not circular. We do not assume the system remains elastic and then "discover" inelasticity from that assumption. Rather, we (i) use an elastic source model only to estimate the pressurisation (or equivalent uplift–pressure relationship) during an interval when seismicity is minor and deformation is approximately linear (our quasi-elastic period), and then (ii) treat that estimate as a baseline rate of elastic energy input to the crust. We then compare this baseline input to an independent observation: the energy released by VT earthquakes, which represents inelastic energy dissipation associated with fault slip and damage (through $\Delta U_e = E_r/\eta$).

  If the system were to remain predominantly elastic, the cumulative elastic strain energy in the crust would continue to increase in proportion to the inferred input rate, and earthquake energy loss would remain small. Instead, we observe intervals where seismic energy release accelerates and eventually becomes comparable to or exceeds the elastic energy input, accompanied by departures from the linear (quasi-elastic) deformation trend. This comparison of an elastic baseline (calibrated in the low-seismicity phase) with independently derived inelastic energy release is what we use to identify the transition to inelastic behaviour.

  We have clarified this in the Results section [lines 223-235].

8. Errors are not discussed. It is well known in the seismic source process literature that seismic efficiency, stress drop, estimates of radiated energy etc can vary by orders of magnitude between individual earthquakes, yet the authors cite none of this literature. Similarly, there are inevitably large errors in the geodetic modeling done here. Error sources need to be very seriously considered in this and all research.

- We have addressed errors in Appendix C in which we quantify and propagate the dominant sources of uncertainty through the full stress-evolution framework using a Monte Carlo approach.

  Specifically, Appendix C accounts for (1) uncertainty in elastic properties by treating the shear modulus G as an effective bulk parameter drawn from a prescribed distribution, reflecting both

measurement uncertainty and the approximation of a layered, heterogeneous crust by a homogeneous elastic model, and (2) uncertainty and event-to-event variability in seismic energy release by explicitly sampling the seismic efficiency η from a log-normal distribution. The log-normal form and parameter ranges are justified using the published compilation of η values for small-magnitude earthquakes [3], and epistemic uncertainty in the distribution parameters is quantified via non-parametric bootstrap resampling.

This approach directly addresses the reviewer's concern that seismic efficiency and radiated energy can vary by orders of magnitude between earthquakes: rather than prescribing a single value, each earthquake is assigned an independent η drawn from a broad distribution, and the resulting variability is propagated through the energy balance and stress calculations. Likewise, uncertainty in geodetic modelling enters through variability in G, which controls the conversion between deformation, stored elastic energy, and inferred stress.

The results of this analysis show that, while these uncertainties affect the absolute magnitudes of inferred elastic strain energy and differential stress, the temporal evolution (including the timing of transitions between quasi-elastic, quasi-inelastic, and inelastic behaviour) is robust across the full ensemble of model realisations (Appendix C; Fig. 12). We have therefore revised the manuscript to emphasise that the method is intended to track relative temporal changes and transition timing rather than absolute stress levels.

We believe this treatment provides a transparent and quantitative assessment of uncertainty that is consistent with the known variability in seismic source processes and geodetic modelling.

9. Line 349: "Since volcanoes primarily deform by changing their shape [2], we assume that the strain energy calculated in Eq. (8) represents the distortional component of strain energy". This doesn't make sense to me. Volumetric changes occur within and above magmatic systems regularly and pore pressures changes are a critical part of the stress regime near volcanoes.

- We agree that volumetric processes occur within and around magmatic systems and can be an important part of volcanic stress regimes. However, our analysis focuses on the partitioning of elastic strain energy stored in the surrounding crust as a result of source pressurisation, rather than focusing on magma or pore-fluid volume changes themselves.

  To clarify this distinction and justify our assumptions, we have substantially expanded the manuscript [lines 420-444] and added a new Appendix A. In the revised text, we explicitly state that ΔV refers to the change in volume of the deforming source (or reservoir), not the volume of magma or pore fluids. The resulting elastic strain energy is stored in the host rock and can be decomposed into volumetric (hydrostatic) and distortional (deviatoric) components.

  Using the penny-shaped crack model, we now explicitly compute both components of elastic strain energy throughout the crust. This analysis demonstrates that, for sill-like sources at both Sierra Negra and Campi Flegrei, the distortional (shear-related) component dominates the elastic strain-energy budget, accounting for approximately 81% and 92% of the total energy, respectively. The volumetric component is spatially diffuse and contributes only a minor fraction of the total stored energy (Appendix A; Fig. A1). Because VT earthquakes at both volcanoes overwhelmingly represent shear failure, and because distortional strain energy is directly linked to deviatoric stress and brittle faulting, we focus on the distortional component as the mechanically relevant energy for interpreting seismicity and crustal failure. This choice does not imply that volumetric deformation or pore-pressure changes are absent, but rather that they do not dominate the elastic energy budget associated with faulting in these systems.

We have revised the main text to clarify these points [lines 420-444] and to explicitly reference Appendix A, which quantitatively supports the assumption that distortional strain energy is the dominant contribution for the source geometries considered.

10. The examples given are not adequate to support the method. It would be better to apply this at volcanoes that have multiple eruptions and long-time histories of quality seismic and deformation data, as the false positive rate must be assessed to fully support the method, particularly as b-values are heavily weighted in assessing the model in the paper. The authors are using the same data set (earthquake catalog) to both calculate stress changes and assess how good the calculations are. Again, there is a potential for circular reasoning.

- Our intention with this study is not to provide a statistical forecasting framework or to quantify false-positive rates, but rather to introduce and demonstrate a physically motivated, energy-based interpretation of unrest at volcanoes reawakening after extended repose.

  The two case studies were selected deliberately because they represent closed-vent volcanic systems undergoing prolonged unrest after extended repose, rather than frequently erupting or persistently degassing open systems. In such closed systems, pressurisation of a subsurface source is recorded directly in surface deformation, making it possible to reconstruct the transfer and storage of elastic strain energy in the surrounding crust. In contrast, in open systems pressure is efficiently relieved by degassing and eruption, so sustained pressurisation, progressive deformation, and the associated energy balance cannot be resolved; the present framework is therefore not applicable in those settings. These closed-vent conditions are precisely those under which eruption forecasting is most challenging and societally relevant, because unrest may persist for years to decades before failure. Calderas were chosen because their unrest episodes typically evolve over long timescales and at relatively low strain rates, allowing the progression from quasi-elastic to inelastic behaviour to be resolved in detail. In many stratovolcanoes, analogous processes likely occur but are compressed in time and may be difficult to observe with currently available geodetic and seismic data.

  We agree that assessing false-positive rates and applying the framework across many volcanoes with multiple eruption cycles would be a valuable next step. However, this requires the analysis of long, homogeneous records of high-quality deformation and seismic data that are currently available for only a limited number of systems, which is beyond the scope of this study.

  Contrary to the reviewer's concern, b-values are not used to assess the quality of the stress model, nor are they used to define the stress evolution. The identification of quasi-elastic, quasi-inelastic, and inelastic regimes is based entirely on the relative rates of energy input (from pressurisation) and energy loss (through seismicity). Changes in b-value are analysed a posteriori as an independent descriptor of earthquake population characteristics and are used only to interpret how faulting behaviour evolves as the system transitions between regimes. They provide contextual support for the inferred mechanical evolution but do not enter the calculations themselves. We further emphasise that the inferred stress evolution does not depend on the observed changes in b-value. The energy-balance framework could yield the same temporal evolution of stored energy and mean differential stress even if the earthquake population exhibited different b-value behaviour. In this sense, b-values neither drive nor constrain the stress calculations. Instead, they provide an independent line of evidence describing how fault populations evolve as deformation progresses. The consistency between b-value trends and the independently inferred stress evolution therefore strengthens the physical interpretation but is not required for it.

11. Throughout the manuscript, except for one section of the methods, "stress" is discussed as if it is

a scalar. Stress is a tensor and the type of stress and the surfaces on which that stress acts needs to be defined specifically throughout the manuscript. The potential interaction between inferred stresses and a regional stress field also deserves a mention. Finally, "Stress" and "strain" are used in a way that reads in portions of the paper as if they're interchangeable, and they are not.

- We now define explicitly that the quantity tracked in this study is a scalar, volume-averaged measure of differential (deviatoric) stress, which we denote as the mean differential stress, σ_d. This definition is introduced in the Introduction and is used consistently throughout the manuscript whenever referring to the volume-integrated stress metric inferred from the energy balance. Where tensor quantities are required (e.g., the principal stresses σ_1, σ_2 and σ_3 and the strain-energy–stress relations), this is now stated explicitly in the Methods and Appendices, and we avoid using the word "stress" without qualification.

  We also address the reviewer's point regarding regional stresses directly in the Methods by writing the energy balance in a general form that includes a regional tectonic loading term, U_r, and then stating explicitly that $U_r = 0$ for the case studies analysed here because regional deformation is negligible relative to volcanic forcing. We further note that the formulation remains general and that regional loading can be incorporated in a straightforward manner by retaining the U_r term.

  Regarding the comment on stress and strain being used interchangeably, we may have missed the specific passages the reviewer had in mind, as no examples were indicated. On re-reading the manuscript, we find that the term "strain" is used only where deformation or elastic strain energy is defined within linear elasticity, and it is not used as a substitute for stress. Nevertheless, we have reviewed the manuscript to ensure that stress and strain terminology is used consistently and unambiguously throughout.

12. Several statements in the introduction simplify the state of eruption forecasting based on seismicity to the point of being incorrect. Because most eruptions occur at open system volcanoes (the frequently erupting volcanoes), most eruptions do not have earthquakes that accelerate into eruption. Also it has long been known that a transition from VT earthquakes to tremor and long period earthquakes is critical for eruption forecasting – a much more nuanced analysis than what can be done with earthquake catalogs alone. Earthquakes accelerate before some eruptions, but it is certainly not all and not the majority.

- We clarify that the issues raised here by the reviewer pertain primarily to eruption forecasting at open-vent, frequently erupting systems, which are explicitly not the focus of this study. The framework presented is designed specifically for closed-vent volcanic systems reawakening after extended repose, where sustained pressurisation, progressive deformation, and prolonged VT seismicity can be resolved [11–14] . We do not claim, nor intend, that the method is applicable to open-system volcanoes, where pressure is efficiently relieved by degassing and eruption and where the assumptions underlying our approach are not satisfied.

  We agree that at many open-system volcanoes, eruptions are preceded by little or no acceleration in VT seismicity, and that transitions from VT earthquakes to tremor and long-period events play a critical role in eruption forecasting. These signals are commonly associated with fluid movement and conduit processes. However, these phenomena address a different physical regime and a different forecasting objective from that considered here. The aim of this study is not to capture the final magma-transport phase of eruption onset, but to diagnose the mechanical evolution of the crust during prolonged unrest in closed systems that occurs before this stage of unrest, using VT seismicity and deformation as indicators of stress accumulation and crustal weakening. Accordingly, the absence of tremor or LP events from our analysis is intentional and reflects the scope of the

study, not an oversight. VT seismicity is treated here as an independent and complementary diagnostic tool within the broader forecasting toolbox, rather than as a universal or sufficient precursor to eruption.

We have revised the Introduction to clarify this scope explicitly and to avoid any implication that the behaviour described here applies to open-vent systems or to eruption forecasting more broadly.

13. The point above is particularly true for caldera systems which are known to have complex precursory sequences with multiple swarms before erupting (see work by Acocella and Newhall). Also caldera systems (including Campi Flegrei) after have very active hydrothermal systems. These have their own seismicity which reflects shallow hydrothermal processes rather than deep intrusion.

- We agree that caldera systems can exhibit complex precursory sequences, including multiple seismic swarms, and that in systems such as Campi Flegrei some portion of the shallow seismicity may reflect hydrothermal processes. Our framework is intended to capture the first-order mechanical evolution of unrest through an energy-balance approach, and does not require seismicity to evolve in a specific temporal style. In particular, the analysis is based on the combined evolution of surface deformation and cumulative seismic energy, and is therefore applicable to both swarm-dominated and more continuous patterns of seismicity. We do not exclude a contribution from hydrothermal processes at Campi Flegrei; rather, our results show that the main temporal transitions identified in the paper can be interpreted without needing to invoke changes in the hydrothermal system as the primary control. The fact that the same framework captures comparable transitions at both Campi Flegrei, where hydrothermal activity is important [15], and Sierra Negra, where such effects are much less developed [16], suggests that it is resolving the first-order processes associated with pressurisation and crustal damage, while hydrothermal effects represent an additional layer of system-specific complexity.

Reviewer 3:

The major issue with the paper lies in its determination of the different deformation stages (i.e., elastic, inelastic), which relies heavily on the assumption of when the linear stage of uplift begins and ends. Different assumptions could lead to significantly different results. For example, the long-term deformation of Campi Flegrei does not clearly exhibit a stage of uplift with a constant rate. Instead, one could describe the deformation history more accurately using a power law. Therefore, addressing the non-uniqueness of this interpretation is crucial.

Moreover, the authors have not fully accounted for crustal relaxation, the complexities of the magma system, or variations in magma overpressure. Some scientists may argue that the overpressure may not evolve linearly, even with constant magma injection, as assumed in the paper. That said, I do agree with the authors that the final stage of crustal damage—marked by accelerating surface deformation accompanied by increasing seismicity—could signal the formation of a major through-going fracture, which may lead to an eruption. This is a strong and valuable conclusion. Therefore, I recommend that the authors carefully address the uncertainties surrounding different interpretations of deformation patterns. They should evaluate whether the proposed framework remains robust and applicable under alternative scenarios. This would significantly strengthen the validity and utility of their methodology.

- We have added two new sensitivity analyses to explicitly evaluate the robustness of our framework under alternative interpretations of deformation and rheology.

  First, to address the non-uniqueness of the inferred pressurisation history (particularly at Campi

Flegrei) we introduce a new sensitivity analysis (Appendix A) in which we repeat the full source-pressure inversion and energy-balance analysis using alternative uplift parameterisation. Thus, we fit the early uplift history using power-law functions initiated at multiple start dates and compare these with the linear fit adopted in the main text. While different parameterisation affect the absolute magnitude of the inferred stress and stored elastic strain energy, the temporal evolution of stress and the timing of the transitions between quasi-elastic, quasi-inelastic, and inelastic deformation regimes remain robust across all tested scenarios.

To further assess the physical plausibility of the uplift attributed to damage, we additionally compare the geodetic moment associated with the excess uplift to the cumulative seismic moment released over the same intervals. At both Campi Flegrei and Sierra Negra, these moments are comparable by order of magnitude, indicating that the amount of uplift attributed to seismicity-induced damage is physically reasonable and consistent with the observed seismic energy release.

Second, to address the potential role of crustal relaxation and non-elastic behaviour, we add a new appendix (Appendix C) that evaluates the influence of viscoelastic relaxation using a Maxwell rheology. We quantify the importance of relaxation using the Deborah number and emulate first-order viscoelastic stress decay by allowing the fraction of source work stored as elastic strain energy to decay exponentially with the Maxwell timescale. Across a wide and geologically reasonable range of effective viscosities, viscoelastic relaxation reduces the absolute magnitude of inferred stress but does not alter the temporal pattern of stress evolution or the timing of deformation regime transitions identified in the main analysis.

These new analyses demonstrate that although deformation at Campi Flegrei can be interpreted using different uplift parameterisation and rheological assumptions, the proposed framework is robust across these alternative scenarios. In particular, the identification and timing of deformation stages do not depend sensitively on assumptions about linear pressurisation or purely elastic behaviour. The main conclusions of the study therefore reflect robust features of the coupled deformation–seismicity evolution rather than artefacts of a particular modelling choice.

Finally, the deformation volume plays a critical role in determining the magnitude of the estimated energy. I conducted a simple numerical simulation of a magma chamber (long axis = 3000 m, short axis = 600 m, depth = 3000 m, and pressure change = 30 MPa). The resulting total elastic strain energy change in the crust was on the order of 10^15 *J, which appears consistent with the authors' estimations. However, it is unclear whether the simplified estimation used in Equation (1) is valid for different magma chamber geometries. Additionally, the choice of deformation volume is problematic. In real cases, deformation theoretically occurs over an infinite volume, making it challenging to define a finite deformation region. It would be beneficial for the authors to address this issue explicitly or, alternatively, use numerical modeling to benchmark their calculations. This approach would help validate the robustness of their framework across varying chamber geometries and deformation scenarios.*

- We have added a new appendix (Appendix B) in which we benchmark the simplified energy formulation used in Eq. (1) against a spatially resolved elastic strain-energy calculation based on a physically motivated source model.

  We compute the full three-dimensional elastic strain field produced by the inflation of a sill-like source using the Fialko model of a penny-shaped crack and evaluate the associated elastic strain-energy density throughout the surrounding crust. This allows us (i) to quantify how elastic strain energy is partitioned between volumetric and distortional components, and (ii) to explicitly assess the spatial extent over which deformation contributes meaningfully to the total energy budget.

To address the comment that deformation theoretically extends over an infinite volume, we introduce an objective definition of a finite deformation volume based on energy content rather than geometry. By radially integrating the deviatoric strain-energy density, we define a deformation radius enclosing 99% of the total deviatoric elastic strain energy. This radius exceeds the physical size of the source, demonstrating that deformation is distributed over a larger crustal region, while remaining finite and well defined in an energetic sense.

Finally, we compare the total deviatoric strain energy obtained by full spatial integration with the source-work estimate used in Eq. (1). For both Campi Flegrei and Sierra Negra, the two approaches yield energy estimates that agree to within an order of magnitude. This benchmarking demonstrates that the simplified formulation provides a reasonable and physically grounded estimate of elastic strain energy for sill-like sources, despite differences in geometry and the non-local nature of deformation.

Together, these additional analyses show that the energy-balance framework is robust to reasonable variations in source geometry and deformation extent, and that the simplified formulation used in the main text captures the dominant energetic behaviour of pressurisation-driven deformation in the crust.

Line 35: "Increases in VT event rate may thus be driven initially by an increase in stress and later by a decrease in the crust's strength." This statement implies that a drop in crustal strength leads to the generation of earthquakes, which is conceptually incorrect. Instead, the drop in crustal strength is a consequence of earthquakes and fracturing processes, rather than a driving force for seismicity. It would be more accurate to clarify that the weakening of the crust, caused by fracturing and prior seismic activity, facilitates further fault slip and deformation, which may indirectly accelerate seismicity. This distinction is important for accurately describing the relationship between crustal strength and earthquake generation.

- We agree. This is also our view but may have been obscured by ambiguous wording. This has been revised.

References

[1] Hiroo Kanamori. Energy Budget of Earthquakes and Seismic Efficiency. In Roman Teisseyre and Eugeniusz Majewski, editors, *Earthquake Thermodynamics and Phase Transformations in the Earth's Interior*, volume 76, chapter 11. International Geophysics, 2001.

[2] CY King. Seismic Efficiency. *Journal Geophysical Research*, 74(6):1702–1703, 1969.

[3] A McGarr. Some Comparisons between Mining-induced and Laboratory Earthquakes. *Pure and Applied Geophysics*, 142(3-4):467–489, 1994.

[4] Michele Dragoni and Cecilia Magnanensi. Displacement and stress produced by a pressurized, spherical magma chamber, surrounded by a viscoelastic shell. *Physics of the Earth and Planetary Interiors*, 56:316–328, 1989.

[5] Matthew Head, James Hickey, Joachim Gottsmann, and Nicolas Fournier. The Influence of Viscoelastic Crustal Rheologies on Volcanic Ground Deformation: Insights From Models of Pressure and Volume Change. *Journal of Geophysical Research: Solid Earth*, 124(8):8127–8146, 8 2019.

[6] Matthew Head, James Hickey, Joachim Gottsmann, and Nicolas Fournier. Exploring the Impact of Thermally Controlled Crustal Viscosity on Volcanic Ground Deformation. *Journal of Geophysical Research: Solid Earth*, 126(8), 8 2021.

[7] Patricia M Gregg, Yan Zhan, Falk Amelung, Dennis Geist, Patricia Mothes, Seid Koric, and Zhang Yunjun. Forecasting mechanical failure and the 26 June 2018 eruption of Sierra Negra Volcano, Galápagos, Ecuador. *Science Advances*, 8(22):eabm4261, 2022.

[8] R. Castaldo, L. D'Auria, S. Pepe, G. Solaro, V. De Novellis, and P. Tizzani. The impact of crustal rheology on natural seismicity: Campi Flegrei caldera case study. *Geoscience Frontiers*, 10(2):453–466, 3 2019.

[9] A. Gudmundsson. Strengths and strain energies of volcanic edifices: Implications for eruptions, collapse calderas, and landslides. *Natural Hazards and Earth System Science*, 12(7):2241–2258, 2012.

[10] A. Gudmundsson. *Volcanotectonics: Understanding the structure, deformation and dynamics of volcanoes*, volume 1. Cambridge University Press, 1 edition, 2020.

[11] Barry Voight. A method for prediction of volcanic eruptions. *Nature*, 332(6160):125–130, 1988.

[12] Christopher R.J. Kilburn. Forecasting Volcanic Eruptions: Beyond the Failure Forecast Method. *Frontiers in Earth Science*, 6(1):133, 9 2018.

[13] Randall A. White and Wendy A. McCausland. A process-based model of pre-eruption seismicity patterns and its use for eruption forecasting at dormant stratovolcanoes. *Journal of Volcanology and Geothermal Research*, 382:267–297, 9 2019.

[14] Reinold R Cornelius and Barry Voight. Seismological aspects of the 1989-1990 eruption at Redoubt Volcano, Alaska: the Materials Failure Forecast Method (FFM) with RSAM and SSAM seismic data. *Journal of Volcanology and Geothermal Research*, 62:469–498, 1994.

[15] G. Chiodini, S. Caliro, C. Cardellini, D. Granieri, R. Avino, A. Baldini, M. Donnini, and C. Minopoli. Long-term variations of the Campi Flegrei, Italy, volcanic system as revealed by the monitoring of hydrothermal activity. *Journal of Geophysical Research: Solid Earth*, 115(3), 3 2010.

[16] F Goff, G M McMurtry, and D J Councé A Simac A R Roldµn-Manzó D R Hilton. Contrasting hydrothermal activity at Sierra Negra and Alcedo volcanoes, Galapagos Archipelago, Ecuador. Technical report, 2000.

Referee response II: Crustal weakening before rupture at volcanoes after long repose

Eric Newland & Christopher Kilburn

Thank you to all the reviewers for their thoughtful and constructive comments. We have addressed each point as fully as possible and believe that the manuscript has been substantially improved as a result. The principal revisions include moving all appendices to the Supplementary Information, in accordance with the journal's formatting requirements; adding a new supplementary section that provides a more detailed description of how the end of the quasi-elastic regime was determined; and softening the language throughout the manuscript. In particular, we now refer to the progression towards rupture rather than towards eruption, recognising that rupture does not necessarily result in an eruption. We have also revised the title of the manuscript to reflect this change in emphasis.

Reviewer 3:

1. *Appendix B figure is labelled "Figure 11" but referred to as "Fig. D1a/b" in the text — reconcile.*

   - Done

2. *Viscosity range is inconsistent: text says* 10^{19}–10^{21} *Pa s, Fig. 14 caption says* 10^{18}–10^{21}

   - Done

3. *A few typos remain (e.g., there are two "robust" in Line-280 and "propagarte" in Line-599).*

   - Done

Reviewer 4:

Line 17-19: A little too colloquial for my taste, but it's up to you.

- We have removed the quotation marks and rephrased the sentence as follows:

  Unrest is therefore accompanied by an increase in fracturing, raising two key questions: will this fracturing lead to rupture of the shallow crust, and will such rupture lead to an eruption?

Line 21: What other signals? Cast somewhat differently, changes in VT rates are common during unrest, but it is hard to know in the moment what the VT rate (or energy release) is that will lead to an eruption.

- We have removed the comparison with other monitoring signals and revised the text as follows:

  Changes in local VT seismicity are commonly used to address these questions because they are readily detected, provide continuous records, and frequently show abrupt increases in event rate during the final approach to eruption.

Line 24: Grouping reference 8 in with deterministic modelling is a stretch

- Thank you for pointing this out. We agree and have moved the reference to the following sentence:

  The crust extends above the pressure source, producing detectable ground deformation and small, local earthquakes, or volcano-tectonic (VT) events [3–6].

Line 74: "rapid increase of 1.77 m until June 2007". Is there an error in units here, or maybe "uplift" should be used instead of "increase"? 1.77m from what?

- Text revised appropriately.

  "Following subsidence during the 2005 eruption, surface uplift began in October 2005. The maximum uplift was recorded at the centre of the caldera at station GV04 [1]. Rapid uplift of 1.77 m occurred between October 2005 and June 2007, after which uplift continued at a steady rate of about 0.3 m yr^{-1} until 2016 (Fig. 1)."

Line 79-81: I assume to get a location you need more than these two stations, so I'm not sure why they are being called out here. Or maybe these are actually event counts (the dN/dt in Figure 1)?

- These two stations were used to determine the event counts at Sierra Negra. Event locations, shown in Fig. 2, are available only from 2018 onwards, following the deployment of a more complete seismic network. We have revised the text to clarify that these stations are mentioned because they were used to determine the event counts.

  "Fig. 2 shows the volcano-tectonic (VT) seismic event rate recorded by Station GS09 (2009-2011) and VCH1 (2012-present), installed on the north rim of the summit caldera in 2012 as part of the new monitoring network (Fig. 2; [1, 2])."

Line 82: Bell uses Mc=1.2 by my reading of the paper.

- [1] plot seismic events with $M > 0.8$ in Fig. 2 and with $M > 1.2$ in Fig. 3. However, in [3], only events with $M > 1.4$ are plotted. We therefore adopted $M_c = 1.4$ as the most conservative estimate of the magnitude of completeness. We do not expect this choice to affect our results substantially, as the changes in seismic energy at later times are dominated by events with $M > 3$.

Line 83-85: The rates appear to be much lower than 830 events/month and seem to be accelerating through 2017 and 2018.

- Thank you for pointing out the inconsistency in this description. We have revised the text accordingly.

  Line 83: "The rate began to accelerate rapidly in 2017 and reached approximately 500 events per month by 2018. It then continued to increase more gradually until the eruption began on 26th June."

Line 152: Apologies if I'm being dense here, but I've read the methods and the main text several times and can't figure out how the b-value is incorporated into the model.

- Thank you for highlighting this point. The *b*-values are not incorporated into the model. Instead, we use the event catalogues, including the event magnitudes, to estimate the energy released by individual seismic events. In the Discussion, we then qualitatively compare the evolution of the mean differential stress with the observed evolution of the *b*-values. We have revised the text here to clarify that the seismic energies are estimated directly from the event magnitudes.

  "Elastic strain energy dissipated by earthquakes is estimated using the event magnitudes together with an empirically constrained seismic efficiency (Methods)"

Line 201: If there were station outages at SN, how do you know that the VT seismicity was low during the initial adjustment phase? Is it worth noting that the maximum magnitude is trending generally higher during this time?

- As the reviewer notes, we cannot directly observe the VT event rate during periods of station outage. However, we assume that the initial post-eruptive resurgence from 2006 to 2010 was predominantly elastic and therefore accompanied by relatively little seismicity, consistent with other observed periods of post-eruptive resurgence [4]. We further assume that the seismicity rate increased approximately monotonically through time, without large, unpredictable variations entirely unrelated to uplift. On this basis, we infer that the rates during station outages were similar to those observed immediately before and after each outage, which remained low between 2010 and 2015. We therefore do not expect unrecorded seismicity during these outages to have substantially affected the inferred evolution of stored elastic strain energy at Sierra Negra. We have revised the text to make these assumptions explicit.

  "At both calderas, deformation begins with an initial adjustment phase (Sierra Negra, 2006–2008; Campi Flegrei, 2005–2011; Fig. 1), followed by a prolonged interval of near-constant uplift rate (Sierra Negra, 2008–2015; Campi Flegrei, 2011–2020; Fig. 1). During these intervals, VT seismicity remains low and event rates increase only gradually. At Sierra Negra, we assume that seismicity rates during outages in the early phases were similar to those recorded immediately before and after each outage and therefore remained low."

Line 208: Based on Figure 1, I don't see a significant difference in the seismicity, as stated in the text, until well after the end of the quasi-elastic phase. Is it worth an inset to show the difference?

Lines 213–214: Figure? This analysis depends greatly on your window size. I don't dispute that there is a change in seismicity eventually, only that it isn't obvious from Figure 1 that the change occurs at the vertical line.

- Thank you for this comment. We have added a short Supplementary Information A describing the analysis in greater detail, together with a new figure showing the sensitivity of the inferred transition dates to the chosen window length. We find that varying the window length between 0.5 and 1.5 years does not substantially affect the inferred transition dates.

Line 216-218: Would love to see a figure of this. At the scale of Figure 1 (and Figure 7), it isn't obvious.

- We have now added insets to Fig. 1 showing the uplift rates. These insets more clearly show the correspondence between changes in uplift rate and VT seismicity rate at both Sierra Negra and Campi Flegrei.

Line 219-222: I'm not sure what this paragraph is referring to. Consider a transition sentence in the paragraph before, or a better topic sentence.

- We have altered the wording of the first sentence to make it clear what we are referring to.

  "The synchronous increases in the rate of uplift and seismicity at both calderas can be explained by two end-member mechanisms: an increase in the rate of source pressurisation or the onset of significant crustal weakening."

Line 280: "robust" is repeated

- Done

Line 284-285: I definitely see the magnitudes increase through the quasi-elastic phase. . . especially at CF. This sentence seems to say two things at once.

Line 330: "constant maximum magnitudes" is disputable.

- Thank you for highlighting this. We have revised the text to describe the evolution of maximum earthquake magnitudes and b-values more accurately.

  "The characteristics of the earthquake populations also show similar temporal variations at Sierra Negra and Campi Flegrei. Maximum earthquake magnitudes increase gradually throughout the period of analysis (Fig. 7). The corresponding seismic b-values (Methods), which describe the frequency-magnitude distributions of earthquakes, show no clear systematic trend until the end of the quasi-elastic regime, after which they decrease steadily towards a near-constant value."

Line 290: No need to use "4.71", nobody knows the magnitude to that precision.

- Done

Line 330: "oscillations in b-value about a stable mean": for SN. . . maybe, but for CF the mean does not have much utility because of how variable the b-value is. The use of "oscillations" and "stable" in the same sentence is also confusing.

Lines 332–334: I like this interpretation a lot, but while the b-values were stable, in part because of the number of events, they do not appear to be meaningfully lower than during some periods of the quasi-elastic phase.

- We agree that the terms "oscillations" and "stable mean" were unclear, particularly given the greater variability in b-value at Campi Flegrei. We intended to indicate that b-values varied during the quasi-elastic phase without displaying a clear systematic trend, before transitioning to a regime characterised by a distinct and sustained decrease during quasi-inelastic and inelastic behaviour. We also agree that the later b-values were not necessarily lower than all values observed during the quasi-elastic phase. We have therefore revised the interpretation to emphasise the change in temporal behaviour, rather than the absolute magnitude of the b-values.

  "The evolution from quasi-elastic to inelastic behaviour also coincided with changes in the characteristics of the VT-event population. During the quasi-elastic phase, maximum VT magnitudes increased gradually, whereas b-values varied without a clear systematic trend (Fig. 7). Quasi-inelastic and inelastic behaviour were subsequently marked by continued increases in maximum magnitude, accompanied by a distinct and sustained decrease in b-value before it stabilised at a near-constant value (Fig. 7). These changes during the later stages indicate a progressive increase in both the maximum dimensions of slipping faults, reflected by the increasing earthquake magnitudes, and the relative proportion of larger events, reflected by the decreasing b-values."

Line 341-343: there are a ton of changes in the b-value during all phases except the inelastic phase and thus I don't think it is very good precursor, unless you are suggesting combining it with something else, in which case this sentence needs to be clarified.

- We have softened the language and clarified that this interpretation is based on the sustained decrease in *b*-value in combination with the other contemporaneous signals.

"The sustained decrease in b-value occurred before the transition to inelastic deformation and, as a result, may be a potential precursor to the onset of crustal weakening by fracture."

Line 366: I'm uncomfortable with the use of precursory as it pertains to CF. Is it before the next eruption…yes. But until CF erupts, it is hard to tell whether this is actually precursory activity that portends an eruption or not.

- We agree that the term "precursory" could be interpreted as implying that the observed changes necessarily precede an eruption, which cannot yet be established at Campi Flegrei. We have therefore revised the text to describe these changes as occurring prior to crustal rupture rather than as direct precursors to eruption. We also clarify that, although the transition to inelastic behaviour may indicate the approach to rupture, rupture alone is not sufficient to produce an eruption. An additional condition must be met: the resulting fracture or fault system must be exploited by a pressurised fluid that remains sufficiently driven to reach the surface. The relationship between rupture formation and eruption onset therefore remains an open question beyond the scope of this study.

  "Although this behaviour may indicate the approach to crustal rupture, it does not necessarily constitute a precursor to eruption. For an eruption to occur, the rupture must be exploited by a fluid sufficiently pressurized to reach the surface. The probability that rupture will lead to an eruption thus remains an open question and lies beyond the scope of this study."

Line 371: What about new magma inputs into the system?

- Thank you for raising this point. We have added a new paragraph explaining how changes in the rate of source pressurisation, including those caused by new magma input, could affect the inferred evolution of elastic strain energy. We also refer to Supplementary Information C, where we evaluate the effect of an accelerating pressurisation rate at Campi Flegrei. This analysis shows that, although the absolute values of the inferred stress evolution change, the estimated transition dates remain unchanged. The additional text is provided below:

  "Additional processes may influence the elastic strain energy stored in the crust, potentially altering the progression of fracturing. They include variable rates of source pressurisation, tectonic earthquakes, hydrothermal activity and viscoelastic behaviour of the crust. For example, we have assumed that strain energy is transferred to the crust under an approximately constant rate of pressurisation. The rate of transfer will vary if the rate of pressurisation changes through time (e.g., owing to variations in the supply of pressurizing fluid). However, as shown for Campi Flegrei in Supplementary Information C, although variable rates of pressurisation may alter the magnitude of stress changes in the crust, they are expected to have only a minor influence on when deformation changes from quasi-elastic to inelastic."

Line 371: Have you considered healing of the crust that may occur during repose. Seems like it may have an effect: Thelen et. al, 2010 10.1029/2010GL044194.

- Thank you for this interesting comment and for providing the reference. Our analysis is defined relative to the state of the crust at the onset of unrest and therefore implicitly accounts for any healing that may have occurred since the previous eruption. However, if this method were applied to multiple episodes of unrest at the same volcano, both inter-eruptive healing and residual damage from previous fracturing would be expected to influence the strength of the crust and the subsequent rate of fracture development. A detailed assessment of these competing processes is beyond the scope of the present study.

Line 389: I would challenge the "real-time" applicability here. Precise GNSS locations are hardly real time, as are quality estimates of earthquake magnitude. Deciding when to stop assuming that a linear trend is being followed takes several abnormal GNSS locations. The analyses here require long time windows to generate b-values and rates over, which are slow to change. I don't necessarily dispute the premise of the sentence, only its applicability in real time or even near real time.

- We have removed the claim of real-time applicability.

Line 393: This paper seems relevant: (Hill, 1977) 10.1029/JB082i008p01347.

- We have added this reference into the discussion.

Line 422: is there a reference for this statement?

- We have added further justification for this statement, together with two supporting references [5, 6].

  "Sierra Negra is located within the interior of the northern Nazca Plate [5]. Residual GPS velocities indicate that internal deformation of the plate occurs at a rate approximately three orders of magnitude lower than the local deformation rates associated with volcanic unrest [6]. Therefore, over the timescale of unrest and the spatial scale of the volcanic edifice, the strain associated with regional tectonic deformation is negligible relative to volcanically induced strain."

Line 424: Can you briefly describe how you would take into account the Ur term in an area where tectonics do play a role?

- We have expanded this sentence to clarify that, where tectonic deformation is significant, regional strain rates can be used to estimate the additional elastic strain energy accumulated in the crust and thereby evaluate the U_r term. We have also added a reference to a study in which the elastic strain energy associated with regional tectonic loading is estimated [7].

  "Regional tectonic deformation could nevertheless be incorporated into the framework by using internally generated strain rates associated with regional loading to estimate the additional elastic strain energy accumulated in the crust [7, 8]."

Can the red line in Figure 5,6,7 be made thicker?

- Done

Figure 1: The units on dN/dt are awkward.

- Simplified to [events/30 d.] for clarity.

Figure 4: Consider moving to the methods section?

- For clarity, and to avoid ambiguity regarding the dimensions used in the model, we prefer to retain Figure 4 in the main text alongside table 1.

Figure 5: Colors could be more contrasted to be more easily interpretable. It's also confusing that you are using the same colors for different things in the same figure.

- Thank you for this comment. We have changed the secondary colour to a more vivid green for contrast.

Figure 8: I really like this figure. In c) the way that the swarm-like seismicity is drawn, I would expect an increase in b-value during swarms (smaller fault planes creating lower magnitude earthquakes), which is not obvious in the analyses.

- Thank you for this comment. We expect that short-term fluctuations in b-value associated with individual swarms may be smoothed by the temporal binning used in our analysis. A higher-resolution investigation of b-value variations during individual swarm episodes would therefore be valuable, but is beyond the scope of the present study.

References

[1] Andrew Forbes Bell, Stephen Hernandez, Peter Christopher La Femina, and Mario Calixto Ruiz. Uplift and Seismicity Driven by Magmatic Inflation at Sierra Negra Volcano, Galápagos Islands. *Journal of Geophysical Research: Solid Earth*, 126(7):e2021JB022244, 7 2021.

[2] Alexandra Alvarado, Mario Ruiz, Patricia Mothes, Hugo Yepes, Mónica Segovia, Mayra Vaca, Cristina Ramos, Wilson Enríquez, Gabriela Ponce, Paul Jarrín, Jorge Aguilar, Wilson Acero, Sandro Vaca, Juan Carlos Singaucho, Daniel Pacheco, and Andrea Córdova. Seismic, volcanic, and geodetic networks in Ecuador: building capacity for monitoring and research. *Seismological Research Letters*, 89(2A):432–439, 3 2018.

[3] Andrew F. Bell, Peter C. La Femina, Mario Ruiz, Falk Amelung, Marco Bagnardi, Christopher J. Bean, Benjamin Bernard, Cynthia Ebinger, Matthew Gleeson, James Grannell, Stephen Hernandez, Machel Higgins, Céline Liorzou, Paul Lundgren, Nathan J. Meier, Martin Möllhoff, Sarah Jaye Oliva, Andres Gorki Ruiz, and Michael J. Stock. Caldera resurgence during the 2018 eruption of Sierra Negra volcano, Galápagos Islands. *Nature Communications*, 12(1):1397, 12 2021.

[4] Jean Luc Got, Aline Peltier, David Marsan, Valerie Ferrazzini, Elodie Brothelande, and Aurore Carrier. Pre-Eruptive Damage, Weakening and Magma-Edifice Coupling at Piton De La Fournaise Volcano. *Journal of Geophysical Research: Solid Earth*, 129(4):e2023JB027595, 4 2024.

[5] Robert W Reynolds, Dennis Geist, and Mark D Kurz. Physical volcanology and structural development of Sierra Negra volcano, Isabela Island, Galápagos archipelago. *Geological Society of America Bulletin*, 107(12):1398–1410, 12 1995.

[6] P. Jarrin, J. M. Nocquet, F. Rolandone, H. Mora-Páez, P. Mothes, and D. Cisneros. Current motion and deformation of the Nazca Plate: new constraints from GPS measurements. *Geophysical Journal International*, 232(2):842–863, 2 2023.

[7] Malte J. Ziebarth, Sebastian von Specht, Oliver Heidbach, Fabrice Cotton, and John G. Anderson. Applying Conservation of Energy to Estimate Earthquake Frequencies from Strain Rates and Stresses. *Journal of Geophysical Research: Solid Earth*, 125(8), 8 2020.

[8] A. Gudmundsson. Strengths and strain energies of volcanic edifices: Implications for eruptions, collapse calderas, and landslides. *Natural Hazards and Earth System Science*, 12(7):2241–2258, 2012.
